# Metal/Metal Redox Isomerism Governed by Configuration

**DOI:** 10.1002/chem.202003120

**Published:** 2020-11-09

**Authors:** Stephan Ludwig, Kai Helmdach, Mareike Hüttenschmidt, Elisabeth Oberem, Jabor Rabeah, Alexander Villinger, Ralf Ludwig, Wolfram W. Seidel

**Affiliations:** ^1^ Institut für Chemie Universität Rostock Albert-Einstein-Straße 3a 18059 Rostock Germany; ^2^ Department Life, Light & Matter Universität Rostock Albert-Einstein-Straße 25 18059 Rostock Germany; ^3^ Leibniz-Institut für Katalyse an der Universität Rostock e.V. Albert-Einstein-Strasse 29a 18059 Rostock Germany

**Keywords:** alkyne ligands, bridging ligands, redox chemistry, redox isomerism, regioselectivity

## Abstract

A pair of diastereomeric dinuclear complexes, [Tp′(CO)BrW{μ‐η^2^‐*C*,*C′*‐κ^2^‐*S*,*P*‐C_2_(PPh_2_)S}Ru(η^5^‐C_5_H_5_)(PPh_3_)], in which W and Ru are bridged by a phosphinyl(thiolato)alkyne in a *side‐on* carbon *P*,*S*‐chelate coordination mode, were synthesized, separated and fully characterized. Even though the isomers are similar in their spectroscopic properties and redox potentials, the *like*‐isomer is oxidized at W while the *unlike*‐isomer is oxidized at Ru, which is proven by IR, NIR and EPR‐spectroscopy supported by spectro‐electrochemistry and computational methods. The second oxidation of the complexes was shown to take place at the metal left unaffected in the first redox step. Finally, the tipping point could be realized in the *unlike* isomer of the electronically tuned thiophenolate congener [Tp′(CO)(PhS)W{μ‐η^2^‐*C*,*C′*‐κ^2^‐*S*,*P*‐C_2_(PPh_2_)S}Ru(η^5^‐C_5_H_5_)‐(PPh_3_)], in which valence trapped W^III^/Ru^II^ and W^II^/Ru^III^ cationic species are at equilibrium.

## Graphical abstract

**Diastereomers differ** in physical properties: A case of real metal/metal redox isomerism is observed in a dinuclear complex with a particularly short bridging ligand. The two stereogenic complex moieties at W and Ru, respectively, are linked by an alkyne based phosphine thiolate chelate ligand. Oxidation of the *like* isomer pertains to tungsten, whereas the *unlike* isomer is oxidised at ruthenium. The diastereomers show opposite redox behaviour!

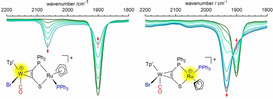

## Introduction

In the last decades valence tautomerism in metal complexes has emerged as a vital research topic in modern coordination chemistry.^[1]^ The term denotes the coexistence of two different isomeric species, which are interrelated by an intramolecular electron transfer. The barrier between both states must be sufficiently high to allow for the spectroscopic characterization of the individual redox forms. The synonymic but more specific term electromerism^[2]^ for the phenomenon takes account of the fact that tautomers as fast interchanging isomers should have different distance matrices, which usually does not apply. The majority of systems, for which the phenomenon is reported so far, involve a redox‐active ligand and a directly coordinated metal. Since the discovery of such metal/ligand valence tautomerism in *ortho*‐benzoquinone complexes of cobalt by Pierpont,^[3]^ new systems with redox‐active ligands based on *ortho*‐quinone,^[4]^ their imino derivatives[^5^, ^6^] or phenolate inclosing Schiff base ligands^[7]^ have been developed. The electronic and magnetic behaviour of their complexes with a variety of transition metals^[8]^ as well as lanthanides^[5]^ has been thoroughly investigated.^[9]^ In particular, switching of magnetic states by external stimuli, like light or heat, has attracted much interest due to potential applications in spintronics and sensing.[^4^, ^10^] However, the limited structural differences of the electromers in bond lengths and angles at the redox centres cause low barriers for the intramolecular electron transfer. The resulting rapid interconversion prevents separation into true redox isomers. Only recently, Himmel and co‐workers presented a dinuclear copper tetrakis(guanidine) complex, which could be obtained in two different redox‐isomeric forms by intentional choice of solvent.^[11]^

Polynuclear complexes, showing metal/metal valence tautomerism, are considerably less well established.^[12]^ Respective dinuclear complexes, in which identical metals in mixed valent states are bridged by symmetric ligands, have been an essential tool for the development of classical electron transfer theory.^[13]^ These basic investigations lead progressively into the topical field of molecular electronics.^[14]^ Related systems with different metals or alternatively an asymmetric bridging ligand can generally exist as metal/metal valence tautomers, if the redox potentials are sufficiently close. Selected examples of this type comprise organometallic compounds with carbon‐based phenylene and/or ethynylene moieties as well as polynuclear complexes relying on bridging cyanide.[^15^, ^16^, ^17^, ^18^] One recently presented Fe(CN)Co(NC)Fe complex can be reversibly switched between Fe^III^‐h.s.‐Co^II^‐Fe^III^ and Fe^III^‐l.s.‐Co^III^‐Fe^II^ (h.s. high spin, l.s. low spin) by energy‐selective irradiation at low temperature.^[18]^ Fundamentally, valence tautomers cannot be separated and isolated in substance. In this contribution we present dinuclear W/Ru complexes, in which two stereogenic metal centres are bridged by a phosphinyl(thiolato)alkyne ligand. The separated diastereomeric complexes form metal/metal redox isomers upon oxidation.

## Results and Discussion

**Scheme 1:**
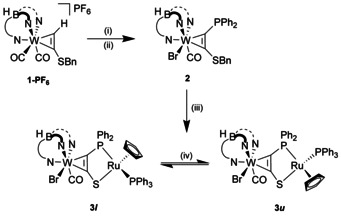
Synthetic pathway to dinuclear complex **3**: (i) *n*Bu_4_Br, THF, rt; (ii) 1) *n*BuLi, −80 °C, THF 2) ClPPh_2_, −80 °C to rt; (iii) 1) [(η^5^‐C_5_H_5_)Ru(PPh_3_)(MeCN)_2_]PF_6_, THF, rt 2) KC_8_, THF, −40 °C to rt; (iv) toluene, 111 °C. For synthetic protocols see Supporting Information.

The synthetic scheme starts with the W^II^ alkyne complex [Tp′W(CO)_2_{η^2^‐C_2_H(SBn)}]PF_6_, **1**‐PF_6_ (Tp′ = hydridotris{3,5‐dimethylpyrazolyl}borate, Scheme 1). After conversion of the complex cation into a neutral one by substitution of a CO ligand by bromide, a phosphinyl group was straightforwardly introduced by deprotonation with *n*BuLi and addition of PPh_2_Cl. According to NMR spectroscopic evidence the alkyne complex **2** exists in two isomeric forms with respect to the alkyne rotation at tungsten. The rotamer mixture shows one distinctive, non‐broadened CO band at 1919 cm^−1^, a difference in the ^31^P chemical shift of less than 1 ppm and a single reversible W^II^/W^III^ oxidation wave at −0.01 V vs. Fc/Fc^+^ in cyclic voltammetry. Isomerization can be observed on a timescale of weeks by NMR monitoring but separation of the two compounds was not pursued, since the mixture proved adequate for the generation of dinuclear compounds. Reaction of complex ligand **2** with [(η^5^‐C_5_H_5_)Ru(PPh_3_)(MeCN)_2_]PF_6_ and subsequent reductive removal of the benzyl group led to the neutral air‐ and water‐stable dinuclear complex [Tp′(CO)BrW{μ‐η^2^‐*C*,*C′*‐κ^2^‐*S*,*P*‐C_2_(PPh_2_)S}Ru(η^5^‐C_5_H_5_)(PPh_3_)] **3**.

**Figure 1:**
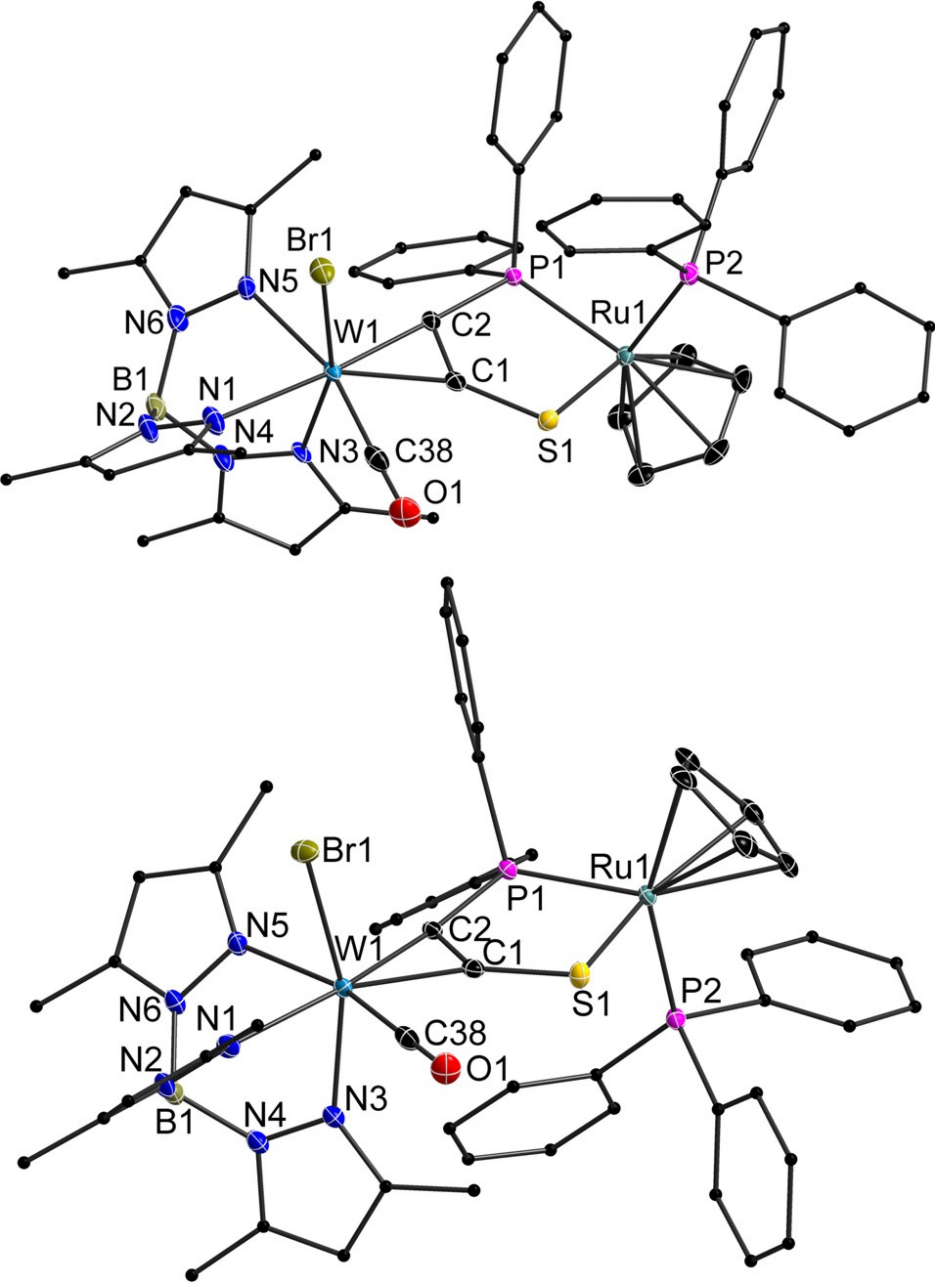
Molecular structure of **3** ***l*** (top) and **3** ***u*** (bottom) in the crystal with ellipsoids set at 50 % probability. Hydrogen atoms have been omitted and pyrazole‐ and Ph‐carbon atoms are depicted as ball‐and‐stick‐models for clarity. Note the relative positions of Br1 and P2. Selected bond lengths [pm] and angles [deg]; **3** ***l***: W1−Br1 258.43(4), W1−C38 194.9(4), W1−N1 228.1(3), W1−N3 218.8(3), W1−N5 227.2(3), W1−C1 204.9(3), W1−C2 202.7(3), C1−C2 134.7(4), C1−S1 169.7(3), C2−P1 182.8(3), S1−Ru1 239.59(8), P1−Ru1 230.41(8), Ru1−P2 230.38(8), C2‐C1‐S1 131.3(2), C1‐C2‐P1 113.1(2), C1‐S1‐Ru1 100.92(11), C2‐P1‐Ru1 106.98(10), S1‐Ru1‐P1 86.10(3); **3** ***u***: W1−Br1 258.42(3), W1−C38 195.6(3), W1−N1 226.1(2), W1−N3 218.6(2), W1−N5 225.5(2), W1−C1 204.2(3), W1−C2 203.6(2), C1−C2 135.0(4), C1−S1 169.5(3), C2−P1 181.9(2), S1−Ru1 238.55(7), P1−Ru1 230.50(7), Ru1‐P2 230.40(7), C2‐C1‐S1 130.9(2), C1‐C2‐P1 111.43(19), C1‐S1‐Ru1 103.15(9), C2‐P1‐Ru1 109.59(9), S1‐Ru1‐P1 84.49(2).

As both metal centres exhibit chirality in their pseudo‐octahedral (W) and pseudo‐tetrahedral (Ru) coordination spheres, two sets of diastereomers were formed, which could be separated by column chromatography and subsequent crystallization. The molecular structures determined by XRD analysis prove the identity as *P*,*S*‐chelate complexes with a diastereomeric relationship (Figure 1). The isomer characterized by the Br‐ligand at W and the PPh_3_ at Ru being oriented towards the same side of the bridging plane shows the same stereodescriptors on both metal centres (*like*) and is therefore denoted as **3** ***l***. Conversely, the second isomer shows an orientation of the Br‐ligand at W and the PPh_3_ at Ru in opposite directions of the plane, leading to different stereodescriptors at the metals (*unlike*) and thereby a notation of **3** ***u*** (see Supporting Information).^[19]^ The pure compounds can be isomerized in refluxing toluene, giving access to increased amounts of the *u*‐isomer. Conveniently, the latter is kinetically less favoured but thermodynamically more stable allowing target‐oriented synthesis of a particular isomer. With the dinuclear compounds **3** ***l*** and **3** ***u*** in hand, we investigated their spectroscopic and chemical properties, especially with regards to configuration‐based differences between the diastereomers.

The bonding parameters in the molecular structures of **3** ***l*** and **3** ***u*** are very similar. Exclusively the distances W1–N1 (*trans* to alkyne), W1–N5 (*trans* to CO) and Ru1–S1 are longer in the *l*‐isomer according to the 3σ significance criterion. The most striking geometrical difference applies to the bend of the C_2_PS bridging moiety, which is more pronounced in the *l*‐isomer (see Supporting Information). Naturally, this bend in **3** ***l*** leads to a shorter W‐Ru distance of 508.5 pm when compared to 515.1 pm in **3** ***u***.

Consistently, in solution the diastereomers show individual sets of ^1^H and ^31^P NMR signals. While the P‐atoms in **3** ***l*** give rise to a single multiplet signal at 50.6 ppm, **3** ***u*** exhibits two sharp doublets at 53.7 and 70.1 ppm, the latter being assigned to the ring P atom. With regard to the electronic structure, the CO stretching frequency in the IR spectrum in CH_2_Cl_2_ differs only by two wavenumbers and the *E*_1/2_ values of the reversible oxidation determined by cyclic voltammetry amount to −0.07 V for **3** ***l*** and −0.04 V for **3** ***u***, respectively. A second oxidation occurs at distinctly higher potentials of +0.55 V (**3** ***l***, partially reversible) and +0.47 V (**3** ***u***, fully reversible). Up to this point, the apparent similarities in electronic behaviour seemed to match the comparable binding parameters of the molecular structures.

However, stoichiometric oxidation of both complexes in CH_2_Cl_2_ produced bewildering results. Addition of acetyl ferrocenium tetrafluoroborate (^Ac^Fc^+^BF_4_^−^, *E*_1/2_ = +0.27 V vs. Fc/Fc^+^)^[20]^ to **3** ***l*** caused both a distinctive colour change from red to green and a drastic shift of Δ*ν*=164 cm^−1^ for the CO stretching frequency. This value is typical for an W^II^/W^III^ oxidation due to the decrease of π‐back‐bonding ability of tungsten. The mononuclear complex **2** shows a change by 168 cm^−1^ upon oxidation, supporting the assignment of a tungsten based oxidation in **3** ***l*** to **3** ***l*** ^+^. Rather unexpectedly, isomer **3** ***u*** shows a similar colour change indeed but a much smaller shift of merely Δ*ν*=28 cm^−1^ in the IR spectrum, indicating a localization of the electron transfer **3** ***u*** to **3** ***u*** ^+^ at ruthenium.

**Figure 2:**
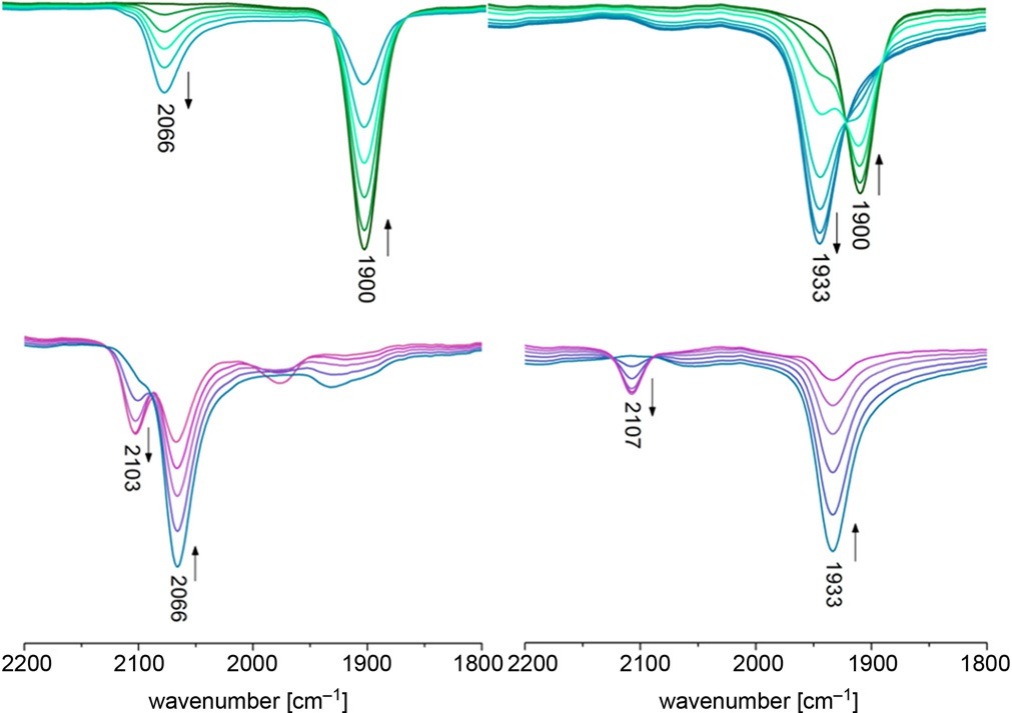
IR‐SEC measurements in 1,2‐dichloroethane (0.26 m *n*Bu_4_PF_6_): redox pairs **3** ***l***/**3** ***l*** ^+^ (top left), **3** ***l*** ^+^ **/3** ***l*** ^2+^ (bottom left) and **3** ***u***/**3** ***u*** ^+^ (top right), **3** ***u*** ^+^/**3** ***u*** ^2+^ (bottom right); small deviations from the wavenumbers reported for the stoichiometric oxidation are due to solvent effects, for those spectra see Supporting Information.

Spectro‐electrochemical investigations (SEC) provided the proof of complementarity of the oxidation steps (Figure 2). Observation of the short‐lived dicationic species **3** ***l*** ^**2+**^ and **3** ***u*** ^**2+**^ at 2103 and 2107 cm^−1^, respectively, indicated now the small change for **3** ***l*** ^+/2+^ {oxidation Ru^II^ to Ru^III^} and the larger shift for **3** ***u*** ^+/2+^ {oxidation W^II^ to W^III^}.

**Figure 3:**
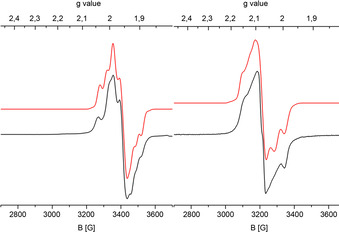
Experimental (black) and simulated (red) X‐band EPR spectra of **3** ***l*** ^+^ (left) and **3** ***u*** ^+^ (right) collected at 100 K in frozen CH_2_Cl_2_.

To confirm the regioselectivity of the oxidation, we recorded X‐band EPR spectra of the mono‐cations (Figure 3). Both W^III^ and Ru^III^ are *S=* ^1^/_2_ ions, which should clearly show different *g* values owing to the differing d‐electrons count (d^3^ and d^5^, respectively).

In frozen CH_2_Cl_2_ solution both cations show rhombic spectra with only partly resolved hyperfine coupling. However, the redox isomers can even already be differentiated by the *g* values. For the W‐oxidized isomer **3** ***l*** ^+^, the main *g* components at 1.921, 1.965 and 1.999 are all smaller than the *g* value of the free electron, while the respective values for the Ru‐oxidized **3** ***u*** ^+^ amount to 2.011, 2.077 and 2.130, matching well with comparable complexes in the literature.[^15^, ^17^, ^21^] The comparatively small *g* value anisotropy in both cases points to substantial delocalization to either bromine or sulfur. Hence, hyperfine coupling to bromine (*S=* ^3^/_2_ for ^79^Br and ^81^Br, combined natural abundance 100 %) is resolved in the spectrum of **3** ***l*** ^+^. In contrast, simulation of the spectrum of cation **3** ***u*** ^**+**^ reveals hyperfine coupling to two ^31^P nuclei of the coordinated phosphine groups.

**Figure 4:**
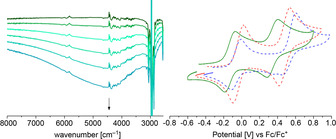
Near‐IR spectrum of **3** ***l*** ^**+**^ (left) collected at rt in 1,2‐dichloroethane during SEC. Cyclic voltammograms (right) of **3** ***l*** (dotted blue), **3** ***u*** (dotted red) and **7** ***u*** (solid green) in CH_2_Cl_2_ with 0.1 m *n*Bu_4_PF_6_ at 0.1 V s^−1^.

The visible absorption spectra of the neutral complexes **3** ***l***/**3** ***u*** and their corresponding cations all exhibit one dominating band, which is characterized by a bathochromic shift of 3790 cm^−1^ for **3** ***l***/**3** ***l*** ^+^ and 4290 cm^−1^ for **3** ***u***/**3** ***u*** ^+^ upon oxidation (see Supporting Information). Interestingly, even though the oxidation is localized at different metal centres, the neutral complexes **3** ***l***/**3** ***u*** show a larger difference of the absorption maxima (680 cm^−1^) compared with the redox isomers **3** ***l*** ^+^/**3** ***u*** ^+^ (180 cm^−1^). Most importantly, both cations show intense intervalence absorptions bands in the near IR region (Figure S4). Electron transfer from Ru^II^ to W^III^ in **3** ***l*** ^+^ is caused by absorption at 2270 nm maximum (4400 cm^−1^, Figure 4) and from W^II^ to Ru^III^ in **3** ***u*** ^+^ by ≈2850 nm excitation (≈3500 cm^−1^).

**Scheme 2:**
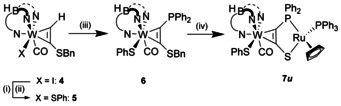
Introduction of the SPh‐ligand and generation of dinuclear complex **7** ***u***. (i) AgOTf, CH_2_Cl_2_/EtOAc, rt; (ii) NaSPh, THF, rt; (iii) 1) *n*BuLi, −80 °C, THF 2) ClPPh_2_, −80 °C to rt; (iv) 1) [Ru(η^5^‐C_5_H_5_)(PPh_3_)(MeCN)_2_]PF_6_, THF, rt 2) KC_8_, THF, −40 °C to rt. For synthetic protocols see Supporting Information.

**Figure 5:**
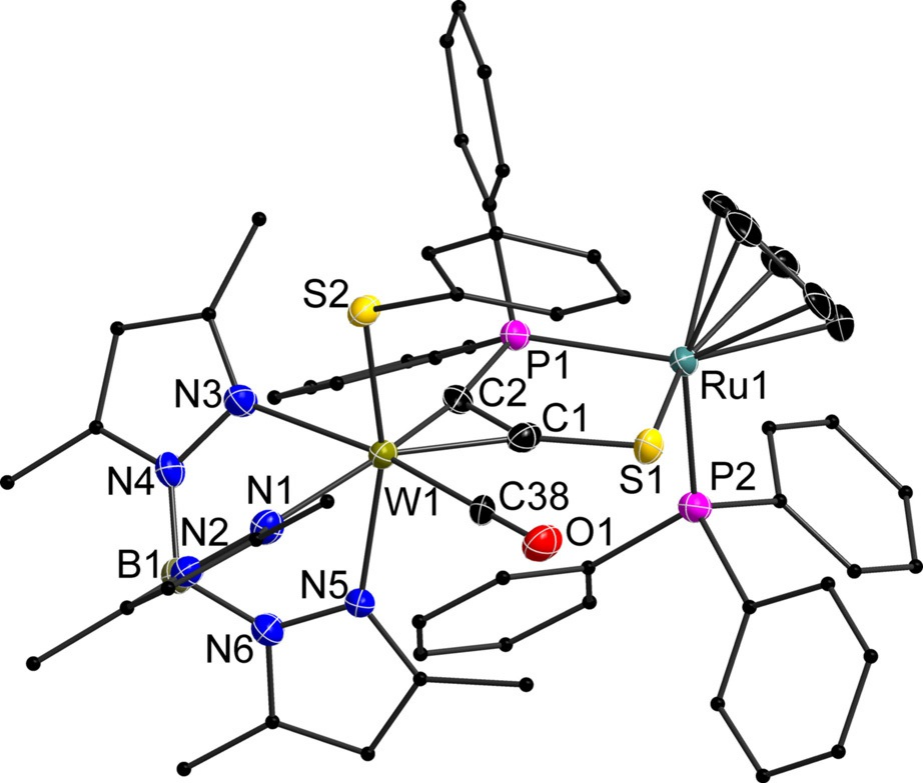
Molecular structure of **7** ***u*** in the crystal with ellipsoids set at 50 % probability. Hydrogen atoms and co‐crystallized solvent have been omitted for clarity. Pyrazole‐ and Ph‐carbons are depicted as ball‐and‐stick‐models. Note the relative positions of S2 and P2. Selected bond lengths [pm] and angles [deg]; W1−S2 238.46(19), W1−C38 196.0(7), W1−N1 228.4(6), W1−N3 228.8(6), W1−N5 223.4(6), W1−C1 206.8(8), W1−C2 202.4(7), C1−C2 133.2(9), C1−S1 169.9(7), C2−P1 182.3(8), S1−Ru1 239.7(2), P1−Ru1 231.7(2), Ru1−P2 230.3(2), C2‐C1‐S1 131.3(2), C1‐C2‐P1 113.1(2), C1‐S1‐Ru1 100.92(11), C2‐P1‐Ru1 106.98(10), S1‐Ru1‐P1 86.10(3).

The clear regioselectivity of the oxidation prompted us to tune the dinuclear system to the tipping point by substitution of co‐ligands. As part of a systematic study, we succeeded in the synthesis of the thiophenolate complex **7** ***u*** (Scheme 2). Since direct substitution experiments at the assembled dinuclear complexes **3** ***l***/**3** ***u*** failed, an alternative reaction sequence had to be developed. The iodide complex **4** could be converted into thiophenolate derivative **5** via an intermediate triflate using consecutively AgOTf and NaSPh. Subsequently, the phosphine function was introduced as described before to yield alkyne complex **6**. Treating complex ligand **6** again with [(η^5^‐C_5_H_5_)Ru(PPh_3_)(MeCN)_2_]PF_6_ and subsequently KC_8_ led to the dinuclear complex **7**. After chromatography, only a single isomer was isolated from this reaction sequence. XRD analysis of suitable crystals uncovered an *unlike* configuration, in which thiophenolate and PPh_3_ are directed at different sides of the planar bridging alkyne ligand (Figure 5). In contrast to **3** ***l***/**3** ***u*** complex **7** ***u*** did not show any isomerization at high temperature.

Advantageously, isomer **7** ***u*** is of particular interest for our investigations, because the diastereomer being characterized by Ru‐oxidation in **3** ***u*** is now substituted at tungsten. Replacement of bromide by the stronger donating thiophenolate should cause a more electron‐rich W complex centre, promoting oxidation at this site.^[22]^ Indeed, the substitution is reflected in most bonding parameters around tungsten. The shorter W1−S2 bond of 238.46(19) pm in **7** ***u*** (compared with 258.42(3) pm for W1−Br1 in **3** ***u***) is accompanied by an elongation of all three W−N bonds, which is most pronounced for W1−N5 in *trans*‐position. In addition, the alkyne is coordinated less symmetrically to tungsten. Surprisingly, the CO vibration in the IR spectrum (being primarily indicative of the electronic situation at W) is not really influenced (Table 1); however the general increase of electron density within the dinuclear complex is reflected by the redox potential. According to cyclic voltammetry, the *E*_1/2_ value has changed about 100 mV from −0.04 V for **3** ***u*** to −0.14 V for **7** ***u*** (Figure 4). A similar shift was observed for the second oxidation at +0.47 V (**3** ***u***) and +0.35 V (**7** ***u***), respectively.

**Table 1:** Comparison of the isomers **3** ***l*** and **3** ***u*** with **7** ***u***.

	**3** ***l***	**3** ***u***	**7** ***u***
X	Br	Br	SPh
colour	red	purple	purple
*δ*(^31^P) PPh_3_ [ppm]	50.6	53.7	54.0
*δ*(^31^P) ring‐P [ppm]	50.6	70.1	67.5
$\tilde{\nu }$_CO_ [cm^−1^]	1903	1905	1906
W−X [pm]	258.43(4)	258.42(3)	238.46(19)
W−N_trans X_ [pm]	218.8(3)	218.6(2)	223.4(6)
*E*_1/2_ (first, second) [V]	−0.07, +0.55	−0.04, +0.47	−0.14, +0.35

**Figure 6:**
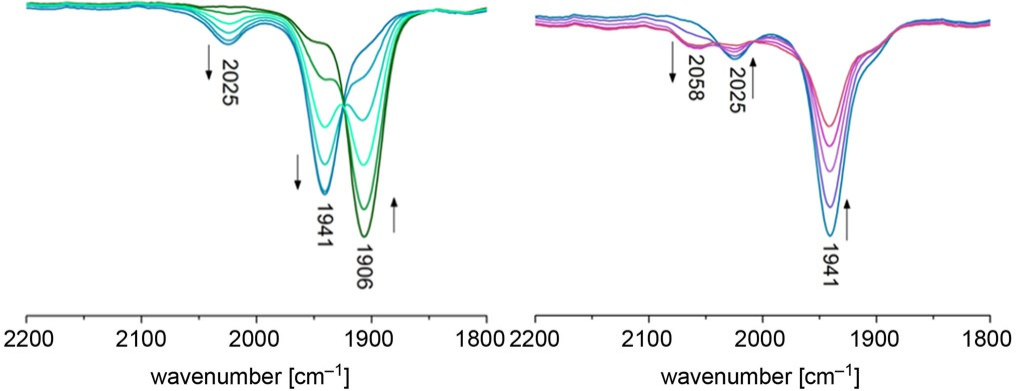
IR‐SEC measurement in 1,2‐dichloroethane (0.26 m *n*Bu_4_PF_6_): redox pairs **7** ***u***/**7** ***u*** ^+^ (left) and **7** ***u*** ^+^/**7** ***u*** ^2+^ (right).

Upon stoichiometric oxidation of complex **7** ***u***, we now observed two distinct new CO absorption bands in the IR spectrum. One stronger band at 1941 cm^−1^ shows a small shift of Δ*ν*=35 cm^−1^ being indicative of a Ru‐centred oxidation. Furthermore a second absorption appears at 2025 cm^−1^, representing a W‐centred oxidation due to a change by 119 cm^−1^. The latter is confirmed by a similar shift of Δ*ν*=128 cm^−1^ for the oxidation of mononuclear SPh‐complex **6** to its cationic form (see Supporting Information). The smaller change in relation to the bromide complexes can be attributed to the charge compensation by the stronger donating thiophenolate. Again, SEC allowed us to observe the dicationic compound **7** ***u*** ^2+^ at 2058 cm^−1^ (Figure 6). Just as with the bromide complexes, this value is close to the sum of the shifts caused by oxidation at each metal.

**Figure 7:**
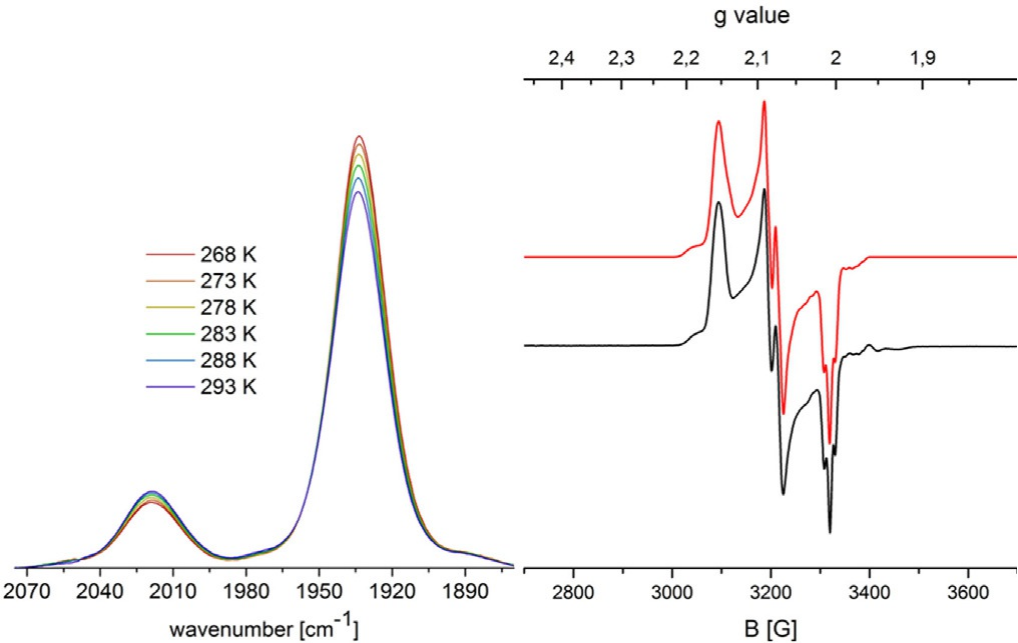
Temperature‐dependent IR‐spectra (left); experimental (black) and simulated (red) X‐band EPR spectrum of **7** ***u*** ^+^ collected at 100 K in frozen CH_2_Cl_2_/ THF mixture (right).

Apparently, both redox forms are now in equilibrium and valence trapped on the IR time scale. Consequently, complex cation **7** ***u*** ^+^ is related to Robin–Day class II for mixed valent complexes, but the redox forms are denoted electromers, because the metal complexes linked are different. Since a solid state IR spectrum of **7** ***u*** ^**+**^ (see Supporting Information) did not show any significant absorption in the region of a W^III^ cation, the Ru^III^ electromer was considered the ground state. To confirm this perception, we collected temperature‐dependent IR spectra of **7** ***u*** ^+^ in CH_2_Cl_2_ solution in the range of −5 °C to 20 °C. Consistently, raising the temperature caused an increase of the higher frequency absorption band associated with the W^III^ species while the lower frequency band being indicative of the Ru^III^ species weakens (Figure 7). The thermodynamic parameters derived from this data are Δ*H*=−1.80 kcal mol^−1^ and Δ*S*=−3.1 cal mol^−1^ K^−1^ for the equilibrium of electromer W^III^Ru^II^‐**7** ***u*** ^+^ with the slightly more stable electromer W^II^Ru^III^‐**7** ***u*** ^+^.

The EPR spectrum of **7** ***u*** ^+^ in a frozen CH_2_Cl_2_/THF mixture at 100 K exhibits a clear hyperfine coupling structure to two different ^31^P nuclei and ^99/101^Ru (*S*=^5^/_2_, combined natural abundance 30 %, Figure 7), which constitutes an unequivocal proof of a Ru‐centred electron spin state at this temperature. In addition, the *g* value set of 2.008, 2.079 and 2.153 is similar to that of **3** ***u*** ^+^ and supports again a Ru^III^ ground state. This observation matches the calculated equilibrium constant of ≈1760 at 100 K for the electromer equilibrium.

For the Br‐complexes, the remarkable dependence of the redox state on the type of the diastereomer is most likely caused by steric interactions of the ligand spheres and a corresponding feedback with the specific complex geometry at the metals. Variation of the anion by using ^Ac^Fc^+^[B{C_6_H_3_(CF_3_)_2_}_4_] instead of ^Ac^Fc^+^BF_4_^−^ as oxidation agent did not result in substantial change of the redox behaviour. Hence, intrinsic structural reasons came into the fore and prompted DFT calculations at the b3lyp/def2‐TZVP/ECP(W,Ru) level of theory. Comparison of the optimized molecular structures of the pairs **3** ***l***/**3** ***l*** ^+^ and **3** ***u***/**3** ***u*** ^+^, respectively, allowed for conclusions on the impact of structural changes at one metal on the overall structure. The calculated metric parameters at the metal centre unaffected by the oxidation match those of the experimentally determined ones of the neutral complex forms in both cases (see Supporting Information). An inherent support for the validity of the calculated cationic species is delivered by TD‐DFT calculations which confirm the assignment of the strong NIR bands to intervalence transfer (see Supporting Information). The calculated and experimental values are in reasonable accordance (**3** ***l*** ^+^: calc. 1685, exp. 2270 nm; **3** ***u*** ^+^: calc. 2992, exp. 2860 nm).

**Figure 8:**
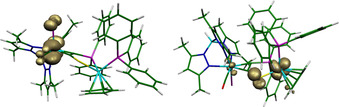
Calculated Mulliken spin densities for a **3** ***l*** ^+^ (left) and **3** ***u*** ^+^ (right).

The frontier orbitals of the neutral isomers **3** ***l***/**3** ***u*** exhibit high similarity showing tungsten centred HOMOs. In contrast, the differing distribution of the Mulliken spin densities of the cations **3** ***l*** ^+^/**3** ***u*** ^+^ reflect the experimental findings (Figure 8). Therefore, specific factors seem to force the Ru‐based oxidation of **3** ***u*** whereas the *like*‐isomer behaves at least qualitatively according to Koopmans’ theorem. Higher calculated reorganization energy after oxidation of **3** ***l*** (0.25 vs. 0.20 eV for **3** ***u***) is accompanied by significant structural changes at tungsten. Essentially, the whole Tp′‐ligand changes its position with respect to the Ru‐complex moiety (B‐W‐Ru angle: 134.2 ° in **3** ***l***; 121.7 ° in **3** ***l*** ^+^). In the *unlike*‐isomer, two of the phenyl rings of different phosphines which are interrelated by π‐stacking are located in one pyrazole‐pocket of the Tp′‐ligand. Accordingly, geometry relaxation after oxidation should be significantly hindered in that isomer, leaving tungsten unable to accommodate the introduced charge. In view of the smaller energy gap between the W‐based HOMO and Ru‐based HOMO‐1 in **3** ***u*** compared with **3** ***l*** (0.24 vs. 0.38 eV), the system can evade this pressure by adapting a Ru^III^ state. Oxidation at ruthenium is particularly characterized by shortening of the Ru−S bond by ≈0.13 Å, which is supported by an experimentally determined value of 0.15 Å for the redox pair [(η^5^‐C_5_H_5_)Ru(dippe)(SPh)]^0/+^ {dippe=bis(diisopropylphosphinyl‐ethan)}.^[23]^ This major change at ruthenium owing to the oxidation should interfere much less with the rest of the molecule (see Supporting Information for a more detailed discussion).

## Conclusion

In summary, on the basis of coordination chemistry with a bridging phosphinyl thiolato alkyne ligand, a first example of diastereomers has been developed, for which the localization of a single redox step belongs to the differences in physical properties. Complex [Tp′(CO)BrW{μ‐η^2^‐*C*,*C′*‐κ^2^‐*P*,*S*‐C_2_(PPh_2_)S}‐ Ru(η^2^‐C_5_H_5_)(PPh_3_)] **3** could be isolated in two stable diastereomeric forms *like* (**3** ***l***) and *unlike* (**3** ***u***), which show similar spectroscopic properties and redox potentials. However, the first oxidation in the *l*‐isomer is assigned to W^II^/W^III^ while a Ru^II^/Ru^III^ redox step is observed in the *u*‐isomer, both processes being reversible. Spectroscopic evidence for this exceptional behaviour was provided by IR, NIR, EPR and spectroelectrochemistry (SEC). Thus, configuration isomerism of a dinuclear complex based on two stereogenic metal centres results in redox regioselectivity. Consistently, the separated oxidation products can be denoted metal/metal redox isomers within a stringent definition. In addition, tuning of the complex system by substitution of the W‐coordinated bromide by thiophenolate allowed the isolation of *unlike*‐[Tp′(CO)(SPh)W{μ‐η^2^‐*C*,*C′*‐κ^2^‐*P*,*S*‐C_2_(PPh_2_)S}Ru(η^5^‐C_5_H_5_)(PPh_3_)] **7** ***u***. This related compound exhibited electromerism, because two valence‐trapped redox forms W^III^Ru^II^ and W^II^Ru^III^ were shown to co‐exist in equilibrium. Ongoing efforts are directed at resolution of the pure enantiomers in order to investigate the redox‐dependence of chiroptic properties.^[24]^ In addition, our findings encourage comprehensive investigations with regard to fine‐tuning by variation of the co‐ligands in the W/Ru complex and to new homoleptic complexes with the *P*,*S*‐alkyne complex ligand and a variety of metals.

## Experimental Section

### Crystallographic data

Deposition numbers 1980458, 1980457, and 1080456 (**3** ***l***, **3** ***u***, and **7** ***u***) contain the supplementary crystallographic data for this paper. These data are provided free of charge by the joint Cambridge Crystallographic Data Centre and Fachinformationszentrum Karlsruhe Access Structures service.

Full experimental details including spectroscopic (^1^H, ^13^C, ^31^P NMR, IR vis/NIR), crystallographic data and details of DFT calculations are given in the Supporting Information.

## Conflict of interest

The authors declare no conflict of interest.

## Supporting information

As a service to our authors and readers, this journal provides supporting information supplied by the authors. Such materials are peer reviewed and may be re‐organized for online delivery, but are not copy‐edited or typeset. Technical support issues arising from supporting information (other than missing files) should be addressed to the authors.

Supplementary
