# Supplementary material for: Metal/Metal Redox Isomerism Governed by Configuration
Source: Chemistry. 2020 Nov 9;26(70):16811–7. doi: 10.1002/chem.202003120 (PMC7756430; doi:10.1002/chem.202003120)
Supplement: Supplementary file 1 — Supplementary [file CHEM-26-16811-s001.pdf]

Chemistry–A European Journal

Supporting Information

Metal/Metal Redox Isomerism Governed by Configuration

Stephan Ludwig,[a] Kai Helmdach,[a] Mareike Hüttenschmidt,[a] Elisabeth Oberem,[b] Jabor Rabeah,[c] Alexander Villinger,[a] Ralf Ludwig,[b] and Wolfram W. Seidel*[a]

1 Contents

1 Contents 1
2 Syntheses 2
2.1 General 2
2.2 $[Tp'W(CO)_2\{\eta^2\text{-}C_2H(SBn)\}]PF_6$, **1**-$PF_6$ 2
2.3 $[Tp'W(CO)Br\{\eta^2\text{-}C_2H(SBn)\}]$ 3
2.4 $[Tp'W(CO)Br\{\eta^2\text{-}C_2(PPh_2)(SBn)\}]$ **2** 3
2.5 $[Tp'(CO)BrW\{\mu\text{-}\eta^2\text{-}\kappa^2\text{-}C_2(PPh_2)S\}Ru(\eta^5\text{-}C_5H_5)(PPh_3)]$ **3** 4
2.6 $[Tp'W(CO)I\{\eta^2\text{-}C_2H(SBn)\}]$ **4** 4
2.7 $[Tp'W(CO)(OTf)\{\eta^2\text{-}C_2H(SBn)\}]$ 5
2.8 $[Tp'W(CO)(SPh)\{\eta^2\text{-}C_2H(SBn)\}]$ **5** 5
2.9 $[Tp'W(CO)(SPh)\{\eta^2\text{-}C_2(PPh_2)(SBn)\}]$ **6** 6
2.10 $[Tp'(CO)(SPh)W\{\mu\text{-}\eta^2\text{-}\kappa^2\text{-}C_2(PPh_2)S\}Ru(\eta^5\text{-}C_5H_5)(PPh_3)]$ **7** 6
2.11 Oxidation of mono- and dinuclear complexes 7
3 X-Ray-Analysis 7
3.1 General 7
3.2 Crystallographic Details for **3*l***, **3*u*** and **7*u*** 8
3.3 Central geometry of **3*l***, **3*u*** and **7*u*** 9
3.4 Determination of Molecular Chirality 9
4 Spectra not shown in main text 10
4.1 UV/VIS spectra of **3*l***/**3*u*** and their cations 10
4.2 NIR spectra of **3*l***$^+$/**3*u***$^+$ 10
4.3 IR-spectra for the chemical oxidation of **2** and **6** 11
4.4 IR-spectra for the chemical oxidation of **3*l***, **3*u*** and **7*u*** 11
4.5 ATR-spectrum of **7*u***$^+$ 12
5 Thermodynamic Parameters of the W(II)Ru(III)-**7*u***$^+$/ W(III)Ru(II)-**7*u***$^+$ equilibrium 12
6 Computation Details 13
7 NMR-Spectra 18
8 References 30
9 Cartesian coordinates of the calculated complexes 31

2 Syntheses

2.1 General

All operations were carried out in an atmosphere of dry argon using Schlenk and glove box techniques. Solvents for reactions were dried and saturated with argon by standard methods and freshly distilled prior to use. Solvents for chromatography were used as purchased from commercial sources. NMR spectra were recorded at 300 K using Bruker Avance 250, 300 or 500 MHz spectrometers. In ^{1}H and ^{13}C NMR, the chemical shifts were internally referenced to the solvent residual peak and in ^{31}P NMR, H_3PO_4 was used as external standard. EPR spectra in X-band were recorded on a Bruker EMX CW-micro spectrometer equipped with an ER 4131VT Digital Temperature Control System and an ER 4119HS-WI high-sensitivity optical resonator. g values have been calculated from the resonance field B_0 and the resonance frequency v using the resonance condition $hv = g\beta B_0$. IR spectroscopy was conducted on a Bruker ALPHA-T (room temperature) and Vertex 80 (temperature-dependent). Elemental analyses were performed with a Thermo Finnigan Flash EA 1112 Series. UV/vis data were collected on an Agilent Cary 60 spectrometer. CV experiments were performed using a Princeton Applied Research VersaSTAT 3 potentiostat. A three-electrode arrangement with a glassy carbon working electrode, a platinum wire counter electrode and a Ag/$AgBF_4$ in CH_3CN reference electrode and 0.1 M $n$$Bu_4NPF_6$ as supporting electrolyte was employed. The Fc/Fc^+ redox couple was used as internal standard. Spectro-electrochemistry was performed in 1,2-dichloroethane with 0.26 M $n$$Bu_4NPF_6$ as supporting electrolyte in an IR cell equipped with silver wire and platinum mesh. The starting materials [Tp'W$(CO)_3$],[1] HC_2SBn,[2] [Ru(η^5-C_5H_5)(PPh_3)$(MeCN)_2$]PF_6[3] and [(η^5-C_5H_5)$_2$Fe]PF_6[4] as well as AgOTf,[5] NaSPh[6] and the oxidizing agent [(η^5-C_5H_5)(η^5-C_5H_4COMe)Fe]BF_4[4] were synthesized according to literature procedures. All other reagents were used as purchased from commercial sources.

2.2 [Tp'W$(CO)_2${η^2-C_2H(SBn)}]PF_6, **1**-PF_6

A stirred solution of 7.44 g (13.17 mmol) [Tp'W$(CO)_3$] and 1.96 g (13.22 mmol) HC_2SBn in 175 mL CH_2Cl_2 was treated with 4.37 g (13.20 mmol) of solid Fc-PF_6. After 30 minutes, CO evolution had ceased and completion of the reaction was confirmed by IR spectroscopy. The solvent was removed in *vacuo* and the remaining dark green solid was left to stand under 80 mL of Et_2O for 30 min to extract most of the ferrocene. After decanting the orange solution, air- and moisture-sensitive **1**-PF_6 was dried in *vacuo* and used without further purification.

IR (CH_2Cl_2): v=2567 (w) (B-H), 2060 (vs), 1986 (vs) cm^{-1} (C-O).

2.3 [Tp'W(CO)Br{η^2-C_2H(SBn)}]

To a stirred solution of 2.94 g (3.54 mmol) **1**-PF_6 in 40 mL THF were added 2.85 g (8.84 mmol) solid *n*Bu_4Br. After a few minutes the end of CO evolution was observed and completion of the reaction was confirmed by IR spectroscopy. The solvent was removed in vacuo and the residue was purified by column chromatography on silica with a mixture of 50 % CH_2Cl_2 and 50 % petroleum ether. After drying, [Tp'W(CO)Br{η^2-C_2H(SBn)}] was obtained as a turquoise powder (1.82 g, 70 %) with both rotamers being present in a non-separable mixture.

major rotamer: ^{1}H NMR (300 MHz, $CDCl_3$): δ=11.96 (s, 1H; C_2H), 7.40-7.25 (m, 5H; Ar-H), 6.08, 5.81, 5.64 (s, 1H; C*H*(CCH_3)$_2$), 4.61 (m, 2H; CH_2), 2.78, 2.55, 2.51, 2.41, 2.32, 1.48 (s, 3H; CH_3); ^{13}C NMR (75 MHz, $CDCl_3$): δ=231.9 (CO), 202.7 (WCH), 199.8 (WCS), 155.0-143.4 (6C, ^{3,5}*C*CH_3), 136.9-127.5 (6C, Ar-C), 108.5-107.0 (3C, ^{4}CH), 44.1 (CH_2), 17.7-12.6 ppm (6C, C*C*H_3); minor rotamer: ^{1}H NMR (300 MHz, $CDCl_3$): δ=12.75 (s, 1H; C_2H), 7.40-7.25 (m, 5H; Ar-H), 6.03, 5.82, 5.69 (s, 1H; C*H*(CCH_3)$_2$), 4.26 (m, 2H; CH_2), 2.84, 2.54, 2.48, 2.42, 2.31, 1.26 (s, 3H; CH_3); ^{13}C NMR (75 MHz, $CDCl_3$): δ=234.7 (CO), 203.9 (WCS), 191.8 (WCH), 155.0-143.4 (6C, ^{3,5}*C*CH_3), 136.9-127.5 (6C, Ar-C), 108.5-107.0 (3C, ^{4}CH), 43.9 (CH_2), 17.7-12.6 ppm (6C, C*C*H_3); mixture of rotamers: IR (THF): ν=2552 (w) (B-H), 1916 (vs) cm^{-1} (C-O); elemental analysis calcd (%) for $C_{25}H_{30}BBrN_6OSW$: C 40.73, H 4.10, N 11.40, S 4.35; found: C 40.84, H 4.24, N 11.19, S 4.24.

2.4 [Tp'W(CO)Br{η^2-C_2(PPh_2)(SBn)}] **2**

A stirred solution of 1.00 g (1.36 mmol) [Tp'W(CO)Br{η^2-C_2H(SBn)}] in 70 mL THF was cooled to – 80 C and treated with 0.71 mL of a 2.5 M solution of *n*BuLi (1.78 mmol) in hexane. After 10 minutes, 0.40 mL (2.17 mmol) $ClPPh_2$ were added and the solution was allowed to warm to room temperature. The solvent was removed in *vacuo* and the residue was purified by column chromatography on silica with toluene. After drying, compound **2** was obtained as a green powder (1.10 g, 88 %) with both rotamers being present in a non-separable mixture.

major rotamer: ^{1}H NMR (250 MHz, $CDCl_3$): δ=8.18-6.75 (m, 15H; Ar-H), 6.03, 5.84, 5.84 (s, 1H; C*H*(CCH_3)$_2$), 4.58 (m, 2H; CH_2), 2.81, 2.56, 2.48, 2.48, 2.34, 1.81 (s, 3H; CH_3); ^{13}C NMR (63 MHz, $CDCl_3$): δ=236.8 (d, $^3J_{C,P}$=5.2 Hz; CO), 210.9 (d, $^2J_{C,P}$=7.5 Hz; WCS), 197.9 (d, $^1J_{C,P}$=44.5 Hz; WCP), 155.0-143.1 (6C, ^{3,5}*C*CH_3), 138.8-127.2 (18C, Ar-C), 108.3-107.2 (3C, ^{4}CH), 40.1 (CH_2), 18.0-12.4 ppm (6C, C*C*H_3); ^{31}P NMR (101 MHz, $CDCl_3$): δ=17.7 ppm (s; WCP); minor rotamer: ^{1}H NMR (250 MHz, $CDCl_3$): δ=8.18-6.75 (m, 15H; Ar-H), 5.97, 5.80, 5.24 (s, 1H; C*H*(CCH_3)$_2$), 3.51 (d, 1H; CH_2), 2.84 (s, 3H; CH_3), 2.73 (d, 1H; CH_2), 2.67, 2.40, 2.30, 2.29, 1.87 (s, 3H; CH_3); ^{13}C NMR (63 MHz, $CDCl_3$): δ=237.2 (d, $^3J_{C,P}$=2.9 Hz; CO), 206.1 (d, $^2J_{C,P}$=3.5 Hz; WCS), 203.8 (d, $^1J_{C,P}$=50.3 Hz; WCP), 155.0-143.1 (6C, ^{3,5}*C*CH_3), 138.8-127.2 (18C, Ar-C), 108.3-107.2 (3C, ^{4}CH), 41.9 (CH_2), 18.0-12.4 ppm (6C, C*C*H_3); ^{31}P NMR (101 MHz, $CDCl_3$): δ=16.8 ppm (s; WCP); mixture of

rotamers: IR (THF): v=2552 (w) (B-H), 1917 (vs) cm^{-1} (C-O); elemental analysis calcd (%) for $C_{37}H_{39}BBrN_6OPSW$: C 48.23, H 4.27, N 9.12, S 3.48; found: C 47.60, H 4.51, N 8.86, S 4.18.

2.5 [Tp'(CO)BrW{μ-η^2-κ^2-$C_2(PPh_2)S$}Ru(η^5-C_5H_5)(PPh_3)] 3

To 285 mg (434 μmol) [Ru(η^5-C_5H_5)(PPh_3)($MeCN)_2$]PF_6 in 15 mL THF was added a solution of 400 mg (434 μmol) **2** and the mixture was allowed to stir over night. The solution was then cooled to –40 °C, 76 mg (562 μmol) KC_8 were added and the mixture was allowed to warm to room temperature and stirred overnight. The solvent was removed in *vacuo* and the residue was purified by column chromatography on silica with CH_2Cl_2. The first, brick red fraction obtained is **3*l*** whereas the purple **3*u*** is eluted subsequently. After drying, the isomers are isolated as reddish solids in a combined yield of 210 mg (38 %). Should a higher ratio of **3*u*** to **3*l*** be required, a portion of **3*l*** can be heated in refluxing toluene for one hour, followed by another chromatography step. Single crystals suitable for XRD were obtained by layering concentrated solutions in CH_2Cl_2 with a tenfold excess of *n*-pentane.

3*l*: ^{1}H NMR (250 MHz, $CDCl_3$): δ=7.43-6.58 (m, 25H; Ar-H), 5.81, 5.71, 5.70 (s, 1H; C*H*($CCH_3)_2$), 4.04 (s, 5H; C_5H_5), 2.77, 2.55, 2.47, 2.26, 2.16, 1.58 (s, 3H; CH_3); ^{13}C NMR (63 MHz, $CDCl_3$): δ=249.3 (m, CO), 230.2 (WCS), 219.1 (d, $^1J_{C,P}$=10.8 Hz; WCP), 155.9, 155.8, 154.6, 145.8, 143.6, 143.4 (^{3,5}$C$$CH_3$), 138.5-126.4 (30C, Ar-C), 108.3, 107.6, 107.6 (^{4}CH), 82.1 (C_5H_5), 17.5, 16.8, 16.7, 12.8, 12.7, 12.7 ppm (C$C$$H_3$); ^{31}P NMR (101 MHz, $CDCl_3$): δ=50.6 ppm (m; WCP, $RuPPh_3$); IR (DCM): v=2555 (w) (B-H), 1902 (vs) cm^{-1} (C-O); elemental analysis calcd (%) for $C_{53}H_{52}BBrN_6OP_2RuSW$: C 50.58, H 4.16, N 6.68, S 2.55; found: C 49.83, H 4.42, N 5.71, S 2.53.

3*u*: ^{1}H NMR (250 MHz, $CDCl_3$): δ=7.37-6.82 (m, 25H; Ar-H), 5.85, 5.65, 5.41 (s, 1H; C*H*($CCH_3)_2$), 4.11 (s, 5H; C_5H_5), 2.69, 2.55, 2.42, 2.21, 1.64, 1.58 (s, 3H; CH_3); ^{13}C NMR (63 MHz, $CDCl_3$): δ=247.3 (d, $^3J_{C,P}$=17.5 Hz; CO), 228.1 (d, $^2J_{C,P}$=5.4 Hz; WCS), 218.1 (d, $^1J_{C,P}$=16.8 Hz; WCP), 160.4, 156.2, 154.1, 146.4, 144.0, 143.1 (^{3,5}$C$$CH_3$), 142.2-127.2 (30C, Ar-C), 109.7, 108.2, 107.4 (^{4}CH), 82.3 (C_5H_5), 16.6, 16.4, 15.9, 12.9, 12.9, 12.7 ppm (C$C$$H_3$); ^{31}P NMR (101 MHz, $CDCl_3$): δ=70.1 (d, $^2J_{P,P}$=43.6 Hz; WCP), 53.7 ppm (d, $^2J_{P,P}$=43.6 Hz; $RuPPh_3$); IR (DCM): v=2558 (w) (B-H), 1905 (vs) cm^{-1} (C-O); elemental analysis calcd (%) for $C_{53}H_{52}BBrN_6OP_2RuSW$: C 50.58, H 4.16, N 6.68, S 2.55; found: C 50.19, H 4.15, N 6.15, S 2.57.

2.6 [Tp'W(CO)I{η^2-$C_2H(SBn)$}] 4

To a stirred solution of 11.75 g (14.15 mmol) **1**-PF_6 in 160 mL THF were added 13.08 g (35.42 mmol) solid $n$$Bu_4$I. After 18 h completion of the reaction was confirmed by IR spectroscopy. The solvent was removed in *vacuo* and the residue was purified by column chromatography on silica. Dark colored byproducts were eluted first with pure CH_2Cl_2. Subsequently a green fraction was collected using a mixture of 50 % CH_2Cl_2 and 50 % petroleum ether. After drying, compound **4** was obtained as a turquoise powder (4.18 g, 38 %) with both rotamers being present in a non-separable mixture.

major rotamer: ^{1}H NMR (250 MHz, $CDCl_3$): δ=12.07 (s, 1H; C_2H), 7.38-7.24 (m, 5H; Ar-H), 6.10, 5.79, 5.62 (s, 1H; C*H*(CCH_3)$_2$), 4.58 (m, 2H; CH_2), 2.79, 2.61, 2.53, 2.39, 2.31, 1.44 (s, 3H; CH_3); ^{13}C NMR (63 MHz, $CDCl_3$): δ=231.2 (CO), 205.8 (WCS), 201.2 (WCH), 155.6-143.5 (6C, ^{3,5}*C*CH_3), 136.7-127.5 (6C, Ar-C), 108.5-106.9 (3C, ^{4}CH), 44.0 (CH_2), 20.0-12.6 ppm (6C, C*C*H_3); minor rotamer: ^{1}H NMR (250 MHz, $CDCl_3$): δ=12.78 (s, 1H; C_2H), 7.38-7.24 (m, 5H; Ar-H), 6.05, 5.80, 5.67 (s, 1H; C*H*(CCH_3)$_2$), 4.22 (m, 2H; CH_2), 2.86, 2.59, 2.50, 2.41, 2.30, 1.24 (s, 3H; CH_3); ^{13}C NMR (63 MHz, $CDCl_3$): δ=233.9 (CO), 205.8 (WCS), 193.0 (WCH), 155.6-143.5 (6C, ^{3,5}*C*CH_3), 136.7-127.5 (6C, Ar-C), 108.5-106.9 (3C, ^{4}CH), 44.0 (CH_2), 20.0-12.6 ppm (6C, C*C*H_3); mixture of rotamers: IR (THF): ν=2555 (w) (B-H), 1919 (vs) cm^{-1} (C-O); elemental analysis calcd (%) for $C_{25}H_{30}BIN_6OSW$: C 38.29, H 3.86, N 10.72, S 4.09; found: C 38.51, H 3.76, N 10.69, S 3.97.

2.7 [Tp'W(CO)(OTf){η^2-C_2H(SBn)}]

To a stirred suspension of 0.50 g (0.64 mmol) **4** in 30 mL EtOAc was added a solution of 0.20 g (0.78 mmol) AgOTf in 4 mL of EtOAc. After five minutes, the solvent was removed in *vacuo* and the residue was purified by column chromatography on silica with CH_2Cl_2. After drying, [Tp'W(CO)(OTf){η^2-C_2H(SBn)}] was obtained as a bright blue powder (0.46 g, 89 %) with both rotamers being present in a non-separable mixture.

major rotamer: ^{1}H NMR (500 MHz, $CDCl_3$): δ=13.04 (s, 1H; C_2H), 7.45-7.26 (m, 5H; Ar-H), 5.97, 5.89, 5.71 (s, 1H; C*H*(CCH_3)$_2$), 4.34 (m, 2H; CH_2), 2.64, 2.48, 2.46, 2.36, 2.19, 1.17 (s, 3H; CH_3); ^{13}C NMR (126 MHz, $CDCl_3$): δ=227.1 (CO), 203.8 (WCH), 201.5 (WCS), 154.4-143.9 (6C, ^{3,5}*C*CH_3), 136.7-127.6 (6C, Ar-C), 118.1 (q, $^1J_{C,F}$=318.8 Hz; CF_3), 108.0-107.3 (3C, ^{4}CH), 44.3 (CH_2), 15.9-12.5 ppm (6C, C*C*H_3); ^{19}F NMR (471 MHz, $CDCl_3$): δ=-76.3 ppm (s; CF_3); minor rotamer: ^{1}H NMR (500 MHz, $CDCl_3$): δ=12.00 (s, 1H; C_2H), 7.45-7.26 (m, 5H; Ar-H), 6.02, 5.88, 5.67 (s, 1H; C*H*(CCH_3)$_2$), 4.69 (brs, 2H; CH_2), 2.59, 2.49, 2.43, 2.37, 2.21, 1.46 (s, 3H; CH_3); ^{13}C NMR (126 MHz, $CDCl_3$): δ=230.4 (CO), 204.9 (WCS), 193.5 (WCH), 154.4-143.9 (6C, ^{3,5}*C*CH_3), 136.7-127.6 (6C, Ar-C), 118.1 (q, $^1J_{C,F}$=318.8 Hz; CF_3), 108.0-107.3 (3C, ^{4}CH), 43.9 (CH_2), 15.9-12.5 ppm (6C, C*C*H_3); ^{19}F NMR (471 MHz, $CDCl_3$): δ=-76.8 ppm (s; CF_3); mixture of rotamers: IR (THF): ν=2555 (w) (B-H), 1933 (vs) cm^{-1} (C-O); elemental analysis calcd (%) for $C_{26}H_{30}BF_3N_6O_4S_2W$: C 38.73, H 3.75, N 10.42, S 7.95; found: C 37.96, H 3.56, N 9.94, S 7.82.

2.8 [Tp'W(CO)(SPh){η^2-C_2H(SBn)}] 5

To as stirred suspension of 0.35 g (2.61 mmol) NaSPh in 40 mL THF was added a solution of 1.62 g (2.01 mmol) of [Tp'W(CO)(OTf){η^2-C_2H(SBn)}] in 20 mL THF. After five minutes, the solvent was removed in *vacuo* and the residue was purified by column chromatography on silica with toluene. After drying, compound **5** was obtained as a dark blue powder (0.76 g, 50 %) with both rotamers being present in a non-separable mixture.

<u>major rotamer</u>: ^{1}H NMR (250 MHz, $CDCl_3$): δ=11.23 (s, 1H; C_2H), 7.45-7.09 (m, 10H; Ar-H), 6.11, 5.73, 5.60 (s, 1H; C*H*(CCH_3)$_2$), 4.57 (m, 2H; CH_2), 2.57, 2.55, 2.40, 2.36, 2.31, 1.71 (s, 3H; CH_3); ^{13}C NMR (63 MHz, $CDCl_3$): δ=234.9 (CO), 190.4 (WCH), 185.5 (WCS), 154.8-143.5 (6C, ^{3,5}*C*CH_3), 137.8-124.5 (12C, Ar-C), 108.6-106.7 (3C, ^{4}CH), 44.1 (CH_2), 17.8-12.6 ppm (6C, C*C*H_3); <u>minor rotamer</u>: ^{1}H NMR (250 MHz, $CDCl_3$): δ=11.98 (s, 1H; C_2H), 7.45-7.09 (m, 10H; Ar-H), 6.09, 5.73, 5.64 (s, 1H; C*H*(CCH_3)$_2$), 4.24 (m, 2H; CH_2), 2.63, 2.52, 2.38, 2.32, 2.29, 1.43 (s, 3H; CH_3); ^{13}C NMR (63 MHz, $CDCl_3$): δ=231.9 (CO), 191.1 (WCS), 176.2 (WCH), 154.8-143.5 (6C, ^{3,5}*C*CH_3), 137.8-124.5 (12C, Ar-C), 108.6-106.7 (3C, ^{4}CH), 43.6 (CH_2), 17.8-12.6 ppm (6C, C*C*H_3); <u>mixture of rotamers</u>: IR (THF): ν=2549 (w) (B-H), 1911 (vs) cm^{-1} (C-O); elemental analysis calcd (%) for $C_{31}H_{35}BN_6OS_2W$·0,5 $C_6H_5CH_3$: C 51.00, H 4.84, N 10.34, S 7.89; found: C 50.98, H 4.93, N 9.75, S 7.90.

2.9 <u>[Tp'W(CO)(SPh){η^2-C_2(PPh_2)(SBn)}]</u> **6**

A stirred solution of 0.76 g (0.99 mmol) **5** in 50 mL THF was cooled to –80 C and treated with 0.52 mL of a 2.5 M solution of *n*BuLi (1.30 mmol) in hexane. After 10 minutes, 0.30 mL (1.63 mmol) $ClPPh_2$ were added and the solution was allowed to warm to room temperature. The solvent was removed in *vacuo* and the residue was purified by column chromatography on silica with toluene. After drying, compound **6** was obtained as a dark green powder (0.82 g, 86 %). By crystallisation from CH_2Cl_2/ pentane, a single rotamer was obtained.

^{1}H NMR (300 MHz, $CDCl_3$): δ=7.58-6.79 (m, 20H; Ar-H), 5.95, 5.66, 5.21 (s, 1H; C*H*(CCH_3)$_2$), 3.95 (m, 2H; CH_2), 2.58, 2.53, 2.30, 2.27, 2.24, 2.24 (s, 3H; CH_3); ^{13}C NMR (75 MHz, $CDCl_3$): δ=233.9 (d, $^{3}J_{C,P}$=2.4 Hz; CO), 193.1 (d, $^{2}J_{C,P}$=4.0 Hz; WCS), 190.0 (d, $^{1}J_{C,P}$=49.0 Hz; WCP), 154.2, 153.0, 151.2, 145.3, 143.5, 143.2 (^{3,5}*C*CH_3), 137.0-124.5 (24C, Ar-C), 108.8, 107.9, 107.4 (^{4}CH), 41.7 (CH_2), 18.3, 18.1, 16.4, 16.3, 12.9, 12.7 ppm (C*C*H_3); ^{31}P NMR (121 MHz, $CDCl_3$): δ=11.4 ppm (s; WCP) IR (THF): ν=2552 (w) (B-H), 1918 (vs) cm^{-1} (C-O); elemental analysis calcd (%) for $C_{43}H_{44}BN_6OPS_2W$·0,5 CH_2Cl_2: C 52.61, H 4.57, N 8.46, S 6.46; found: C 52.44, H 4.76, N 7.95, S 6.66.

2.10 <u>[Tp'(CO)(SPh)W{μ-η^2-κ^2-C_2(PPh_2)S}Ru(η^5-C_5H_5)(PPh_3)]</u> **7*u***

To 160 mg (244 μmol) [Ru(η^5-C_5H_5)(PPh_3)($MeCN$)$_2$]PF_6 in 20 mL THF was added a solution of 250 mg (263 μmol) **6** in 15 mL THF and the mixture was allowed to stir over night. The solution was then cooled to –40 °C, 43 mg (318 μmol) KC_8 were added and the mixture was allowed to warm to room temperature and stirred overnight. The solvent was removed in *vacuo* and the residue was purified by column chromatography on silica using a mixture of 50 % CH_2Cl_2 and 50 % petroleum ether. Compound **7*u*** is eluted as a purple band and is isolated as a reddish solid in a yield of 81 mg (24 %). Single crystals suitable for XRD were obtained by layering a concentrated solution of **7*u*** in CH_2Cl_2 with a tenfold excess of *n*-pentane.

^{1}H NMR (500 MHz, $CDCl_3$): δ=7.70-6.72 (m, 30H; Ar-H), 5.86 (br), 5.57, 5.35 (s, 1H; C*H*(CCH_3)$_2$), 4.08 (s, 5H; C_5H_5), 2.58, 2.40, 2.24, 2.19, 1.65, 1.58 (s, 3H; CH_3); ^{13}C NMR (126 MHz, $CDCl_3$): δ=240.0 (d, $^{3}J_{C,P}$=17.4 Hz; CO), 229.3 (br; WC), 204.5 (br; WC), 159.2, 155.9, 153.8, 145.5, 144.4, 142.7 (^{3,5}*C*CH_3), 134.0-123.6 (36C, Ar-C), 109.4, 108.3, 107.0 (^{4}CH), 82.1 (C_5H_5), 16.4, 15.6, 15.6, 13.1, 12.8, 12.6 ppm (C*C*H_3); ^{31}P NMR (202 MHz, $CDCl_3$): δ=67.5 (d, $^{2}J_{P,P}$=46.7 Hz; WCP), 54.0 ppm (d, $^{2}J_{P,P}$=46.7 Hz; $RuPPh_3$); IR (DCM): v=2557 (w) (B-H), 1906 (vs) cm^{-1} (C-O); elemental analysis calcd (%) for $C_{59}H_{57}BN_6OP_2RuS_2W\cdot CH_2Cl_2$: C 52.49, H 4.33, N 6.12, S 4.67; found: C 52.70, H 4.57, N 5.60, S 4.96.

2.11 Oxidation of mono- and dinuclear complexes

The neutral species **2**, **3**, **6** and **7** were each dissolved in CH_2Cl_2; and the IR spectra were measured. The solvent was then removed in *vacuo* and [(η^5-C_5H_5)(η^5-C_5H_4COMe)Fe]BF_4 was added in a slightly under-stoichiometric amount to prevent excess Fe(III) showing up in the EPR. The solids were then re-dissolved in the same volume of CH_2Cl_2 as before and cooled to –20 °C at which temperature they can be kept for several hours.

3 X-Ray Analysis

3.1 General

Single crystals suitable for X-ray diffraction analysis were selected in Fomblin YR-1800 perfluoropoly-ether oil (Alfa Aesar) at ambient temperature and mounted on a glass fiber. During the measurement, the samples were cooled to 123(2) K. Diffraction data were collected on a Bruker D8 QUEST diffrac-tometer and a Bruker Kappa Apex II diffractometer using graphite monochromated Mo-Kα radiation (λ = 0.71073 Å). Structure solutions were found by direct methods (SHELXS-2014/7) and were refined by full-matrix least-squares procedures on F^2 (SHELXL-2014/7). All non-hydrogen atoms were anisotrop-ically refined unless stated otherwise. Hydrogen atoms were included at calculated positions with fixed thermal parameters unless stated otherwise. The unit cell of **3*l*** contains five dichloromethane mole-cules, which have been treated as a diffuse contribution to the overall scattering without specific atom positions by SQUEEZE/PLATON. The calculated residual electron density of 3.99 eA^{-3} at W1 in **7*u*** is most probably caused by absorption effects.

3.2 Crystallographic Details for **3*l***, **3*u*** and **7*u***

Table S1. Crystallographic details for **3*l***, **3*u***, and **7*u***.

compound	**3*l***	**3*u***	**7*u***
empirical formula	$C_{53}H_{52}BBrN_6OP_2RuSW$ ·1.25(CH_2Cl_2)	$C_{53}H_{52}BBrN_6OP_2RuSW$ ·(CH_2Cl_2)	$C_{59}H_{57}BN_6OP_2RuS_2W$ ·(C_5H_{12})
fw (g mol^{-1})	1364.80	1343.57	1360.04
crystal size (mm)	0.20x0.14x0.07	0.23x0.16x0.06	0.33x0.06x0.06
crystal system	monoclinic	monoclinic	monoclinic
space group	$P2_1$/c	$P2_1/n$	$P2_1/n$
a (Å)	10.5464(5)	12.1144(3)	11.7768(15)
b (Å)	28.3968(15)	10.7740(2)	25.577(3)
c (Å)	17.9316(10)	41.1219(8)	19.974(3)
α (deg)	90	90	90
β (deg)	93.932(2)	96.010(1)	97.432(3)
γ (deg)	90	90	90
V (Å^3)	5357.6(5)	5337.8(2)	5966.1(13)
Z	4	4	4
T (K)	123	123	123
ρ (g cm^{-3})	1.692	1.672	1.514
μ (mm^{-1})	3.44	3.43	2.35
measured reflections	245789	128119	74598
independent reflections	18580	23508	15845
reflections with I>2σ(I)	15580	17156	10259
R_{int}	0.083	0.085	0.126
R1 (F[I>2σ(I)])	0.049	0.047	0.067
wR2 (F^2[all data])	0.078	0.067	0.123
GOF	1.110	1.074	1.088
parameters	610	637	712
CCDC no.	1980458	1980457	1980456

3.3 Geometry of the complex core in **3*l***, **3*u*** and **7*u***

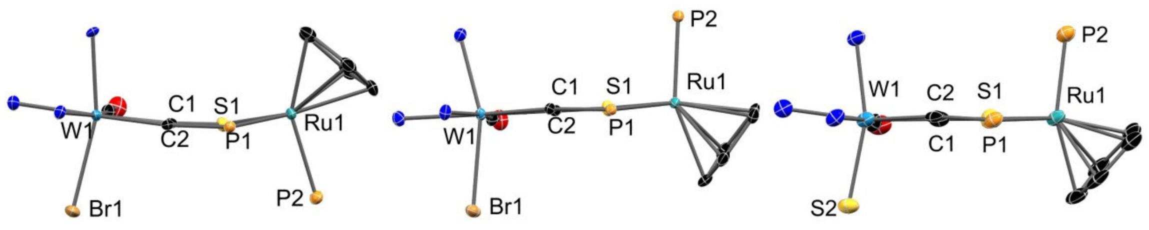

Figure S1. Geometry of **3*l*** (left, W1–Ru1 508.5 pm; W1–C1–C2–Ru1 168.6 °), **3*u*** (centre, W1–Ru1 515.1 pm; W1–C1–C2–Ru1 180.0 °) and **7*u*** (right, W1–Ru1 516.3 pm; W1–C1–C2–Ru1 178.5 °).

3.4 Determination of Molecular Chirality

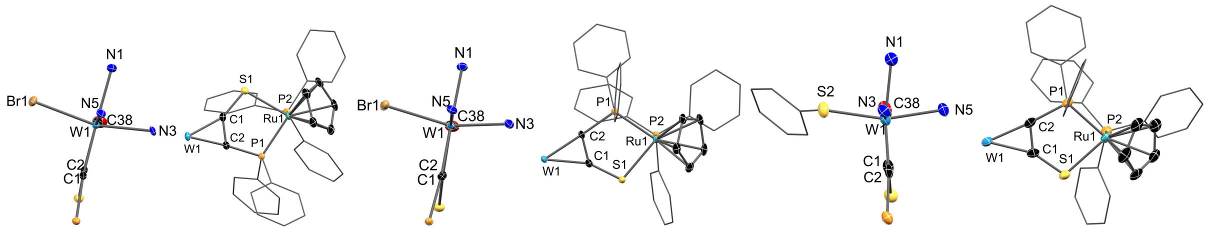

Figure S2. Central ligand spheres around the chiral metal centres in **3*l*** (left), **3*u*** (centre) and **7*u*** (right).

Stereo descriptors have been determined for W and Ru in their respective coordination spheres according to the CIP-rules.[7]

For W, the rules for an octahedral ligand sphere apply, if the alkyne is considered as a single ligand. In such an octahedral complex all N atoms of the tripodal Tp'-ligand are of highest priority. The CO-ligand (lowest priority) and the N atom in *trans* position to CO are then aligned as seen in Figure S2. The remaining four ligands make a cyclic sequence which is "left-handed" in all three complexes, because the higher priority donors N, N are followed by the X ligand, since this has higher priority than the alkyne.

For Ru, the well-known rules for a tetrahedral coordination sphere apply. PPh_3 has lower priority than the PPh_2(CW) moiety while the C_5H_5 ring is treated like a single atom with atomic number 30. This leaves a "left handed" sequence for the W-oxidized isomer of **3** and a "right handed" sequence for the other two complexes.

Thus, both W and Ru show the same stereo descriptors (*like*) in W-oxidized **3*l***, while they do not (*unlike*) in **3*u*** and **7*u***.

4 Spectra not shown in main text

4.1 UV/VIS spectra of **3*l*/3*u*** and their cations

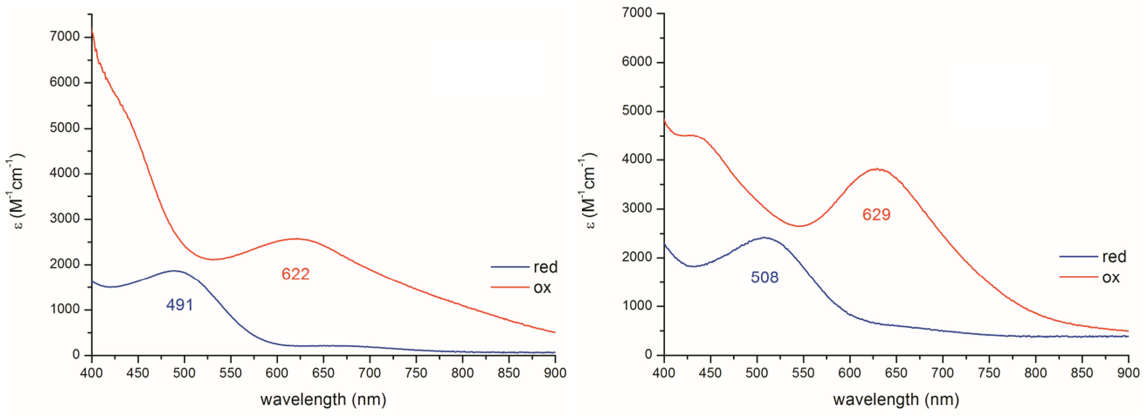

Figure S3. UV/VIS spectra of the neutral (blue) and oxidized (red) states of **3*l*** (left) and **3*u*** (right) collected at ambient temperature in CH_2Cl_2. Acetylferrocene is present after oxidation and absorbs at 453 nm.

4.2 NIR spectra of **3*l*$^+$/3*u*$^+$**

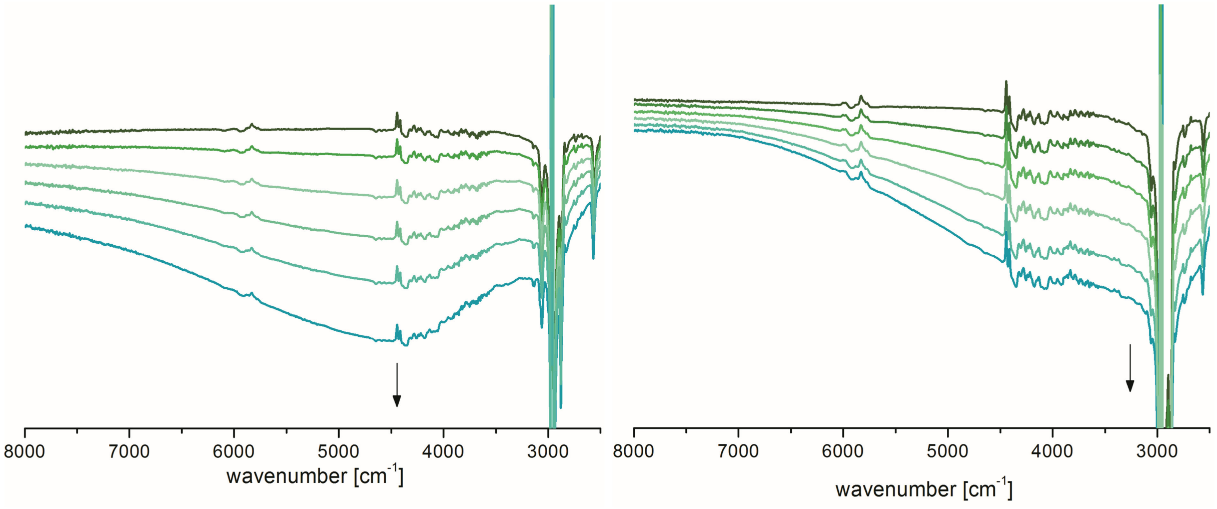

Figure S4. NIR spectra of the cations **3*l*$^+$/3*u*$^+$** in 1,2-dichloroethane as collected during SEC.

4.3 IR-spectra for the chemical oxidation of **2** and **6**

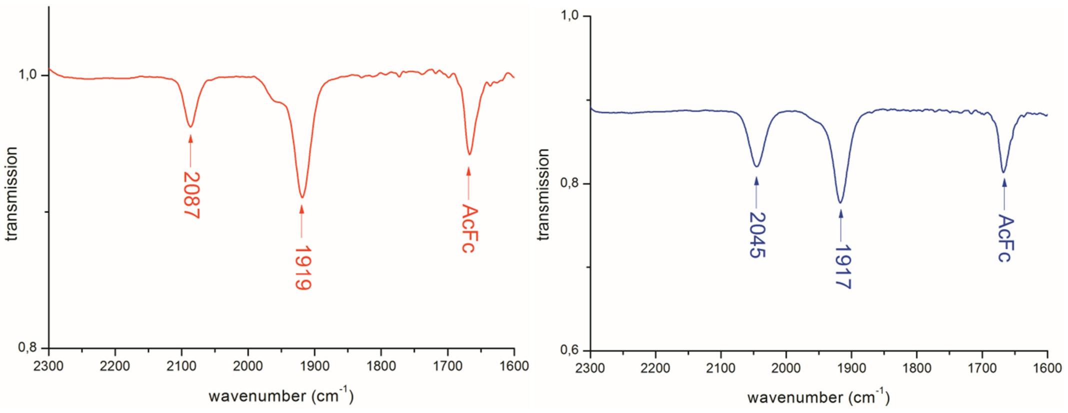

Figure S5. IR spectra of **2** (left) and **6** (right) after addition of an under-stoichiometric amount of [(η^5-C_5H_5)(η^5-C_5H_4COMe)Fe]BF_4. Decomposition at room temperature is indicated by the shoulder at about 1970 cm^{-1}. Note the difference in shift between bromide **2** to **2**$^+$ (168 cm^{-1}) and thiophenolate **6** to **6**$^+$ (128 cm^{-1}).

4.4 IR-spectra for the chemical oxidation of **3*l***, **3*u*** and **7*u***

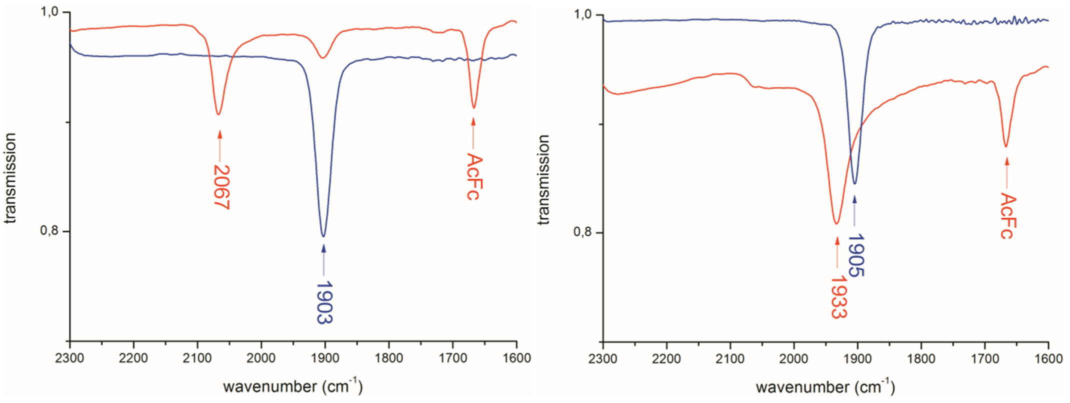

Figure S6. IR spectra of **3*l*** (left) and **3*u*** (right) before (blue) and after (red) addition of a slightly under-stoichiometric amount of [(η^5-C_5H_5)(η^5-C_5H_4COMe)Fe]BF_4.

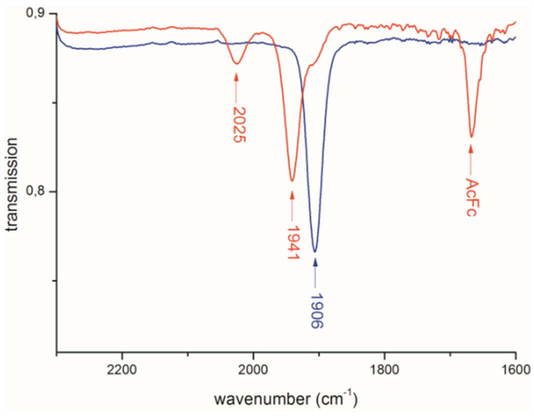

Figure 7. IR spectra of **7*u*** before (blue) and after (red) addition of a slightly under-stoichiometric amount of [(η^5-C_5H_5)(η^5 C_5H_4COMe)Fe]BF_4

4.5 ATR-spectrum of 7*u*$^+$

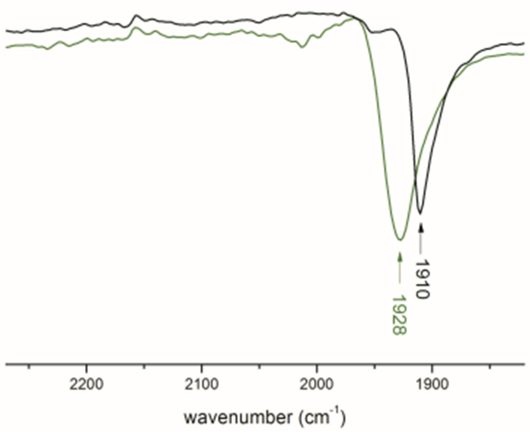

Figure S8. ATR-spectrum of **7*u*** before (black) and after (green) addition of a slightly under-stoichiometric amount of [(η^5-C_5H_5)(η^5-C_5H_4COMe)Fe]BF_4 and removal of solvent.

5 Thermodynamic parameters of the W(II)Ru(III)-7*u*$^+$/ W(III)Ru(II)-7*u*$^+$ equilibrium

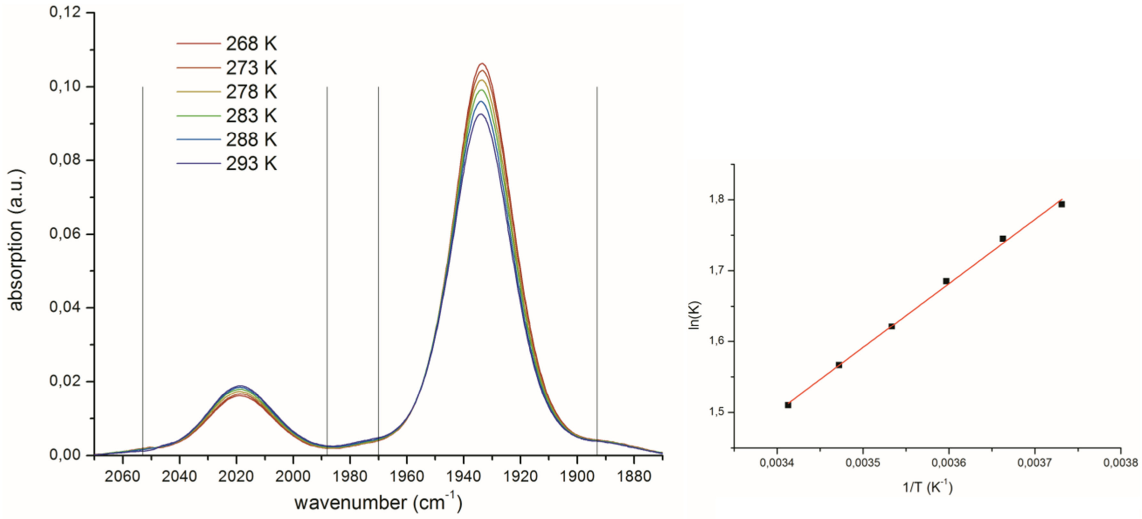

Figure S9. Temperature-dependent IR-spectra of **7u**$^+$ (left) and the van 't Hoff plot derived therefrom (right).

The IR bands for the W(III)-species (higher wavenumbers) and the Ru(III)-species (lower wavenumbers) were integrated within the limits indicated by the straight lines in Figure S9 (left). The baseline between the signals shows a minimum at 1988 cm^{-1} and reaches the same absorption again at 2053 cm^{-1}, the W(III)-signal was therefore integrated within these limits. The Ru(III)-signal shows an approximated saddle point at 1893 cm^{-1}, reaches the same absorption again at 1970 cm^{-1} and was therefore integrated within these limits.

Table S2. Temperature-dependent integrals and equilibrium constants for the **7u**$^{+}$-electromers.

T (K)	integral W	integral Ru	K (Ru/W)	ln(K)	1/T (K^{-1})
268	0,50479	3,03359	6,00960796	1,79335951	0,00373134
273	0,52114	2,98349	5,72492996	1,74483032	0,003663
278	0,54082	2,91616	5,39210828	1,68493646	0,00359712
283	0,56305	2,84827	5,05864488	1,62109864	0,00353357
288	0,57706	2,76332	4,78861817	1,56624189	0,00347222
293	0,59152	2,67762	4,52667704	1,50998812	0,00341297

The equilibrium constants derived from the integrals were then used in a van 't Hoff plot (Figure S9, right), giving a linear fit following the equation $\ln(K) = 904.32(^{1}/_{T}) - 1.57$ with $R^2 = 0.9972$. Multiplication of slope and intercept value with 8.314 J/(mol K) gives $\Delta H = -7.52$ kJ/mol = –1.80 kcal/mol and $\Delta S = -13.09$ J/(mol K) = –3.12 cal/(mol K), respectively.

6 Calculation Details

DFT calculations were carried out using either the G09RevE.01[8] or the ORCA 4.1.1[9] program package. The molecular geometries of complexes **3*l*** and **3*u*** (closed shell singlet) as well as their cations **3*l***$^{+}$ and **3*u***$^{+}$ (open-shell doublet) were optimized without truncation and symmetry constraints in the gas phase using both the B3LYP and PBE0 functional[10] Quasi-relativistic effective core potentials of the Stuttgart/Cologne group were used for W (ECP60MBW) and Ru (ECP28MWB) in combination with a (8s7p6d2f1g)/[6s5p3d2f1g] basis set for both metals.[11] Split valence triple ζ-basis sets (def2-TZVP) of the Ahlrich group were used for the other elements.[12] Dispersion was accounted for by the atom-pairwise dispersion correction with the Becke-Johnson damping scheme (D3BJ).[13] In doing so reasonable matches between the calculated and the experimentally determined structures for the neutral complexes **3*l*** and **3*u*** were achieved. A comparison of calculated and experimentally determined metric parameters for **3*l*** and **3*u*** is given in Table S3. The strongest deviations (1.70 %, 1.45 %) arise for the coordinative bonds W–N, Ru–Cp(centroid) and Ru–S, while most deviations are below 1 %. The molecular structure of the local minimum **3*u***$^{+}$-W(III) was found by deliberate alterations to bond distances in the starting structure of the optimization. The same strategy was not successful for the Ru(III) state of **3*l***$^{+}$. Frequency calculations were performed with a smaller def2-SVP basis set in order to identify all stationary points as minima. The final enthalpies (ΔH) were calculated using the total electronic energy E_{tot} from the higher level calculation and the thermal correction to enthalpy from the frequency calculation. The calculated higher stability of the *unlike* isomer matches the experimental observation of isomerization of **3*l*** to **3*u*** at elevated temperatures. Ionization potentials were deduced from the difference of E_{tot} of the neutral and cationic species based on the optimized geometry of the neutral complex. Reorganization energies λ were inferred from the difference of E_{tot} of the cationic species based on the optimized geometries of the neutral and of the cationic complexes. All-electron

scalar relativistic calculations using ZORA were performed with ORCA as single point calculations. These calculations show that the calculated energy differences between **3*u***$^+$-Ru(III) and **3*u***$^+$-W(III) are smaller than the basis set dependence (Table S4). TD-DFT calculations for complex cations **3*l***$^+$ and **3*u***$^+$ were performed for the first three doublet transitions being free of spin contamination using Gaussian.

Table S3. Comparison of essential bond lengths [Å] for **3*l*** and **3*u*** obtained by X-ray structure analysis and single molecule geometry optimization in the gas phase, the calculated bond lengths were rounded for clarity.

	**3*l*** (exp.)	**3*l*** (calc.)	**3*u*** (exp.)	**3*u*** (calc.)
W–CO	1.949(4)	1.9641	1.956(3)	1.9756
W–Br	2.5843(4)	2.6110	2.5842(3)	2.5952
W–C1 (alkyne)	2.049(3)	2.0567	2.042(3)	2.0580
W–C2 (alkyne)	2.027(3)	2.0293	2.036(2)	2.0305
C1–C2	1.347(4)	1.3483	1.350(4)	1.3486
C1–S	1.697(3)	1.6882	1.695(3)	1.6908
C2–PPh_2 (ring)	1.828(3)	1.8073	1.819(2)	1.8087
W–N (*trans*-C_2)	2.281(3)	2.2917	2.261(2)	2.2994
W–N (*trans*-CO)	2.272(3)	2.3152	2.255(2)	2.2860
W–N (*trans*-Br)	2.188(3)	2.2180	2.186(2)	2.2116
Ru–C_5H_5 (centroid)	1.8768	1.9028	1.8649	1.8970
Ru–S	2.3959(8)	2.4099	2.3855(7)	2.4201
Ru–PPh_2 (ring)	2.3041(8)	2.3147	2.3050(7)	2.2903
Ru–PPh_3 (terminal)	2.3038(8)	2.2942	2.3040(7)	2.2846

Table S4. Calculated total electronic energies E_{tot} and enthapies ΔH in kcal/mol; ionization potential *IP* and reorganization energy λ in eV.

	**3*l***	**3*l***$^+$	**3*u***	**3*u***$^+$-Ru(III)	**3*u***$^+$-W(III)
ΔE_{tot} (b3lyp, ECP)	+4.3	+3.7	0	0	+0.1
ΔH (b3lyp, ECP)	+4.7	+3.8	0	0	–0.3
ΔE_{tot} (PBE0, ECP)	–	+2.6	–	0	+1.0
ΔE_{tot} (PBE0, ZORA)[a]	–	+2.5	–	0	+1.0
IP/λ	5.68/0.25		5.65/0.20		

[a] single point calculation based on the PBE0/ECP molecular structure

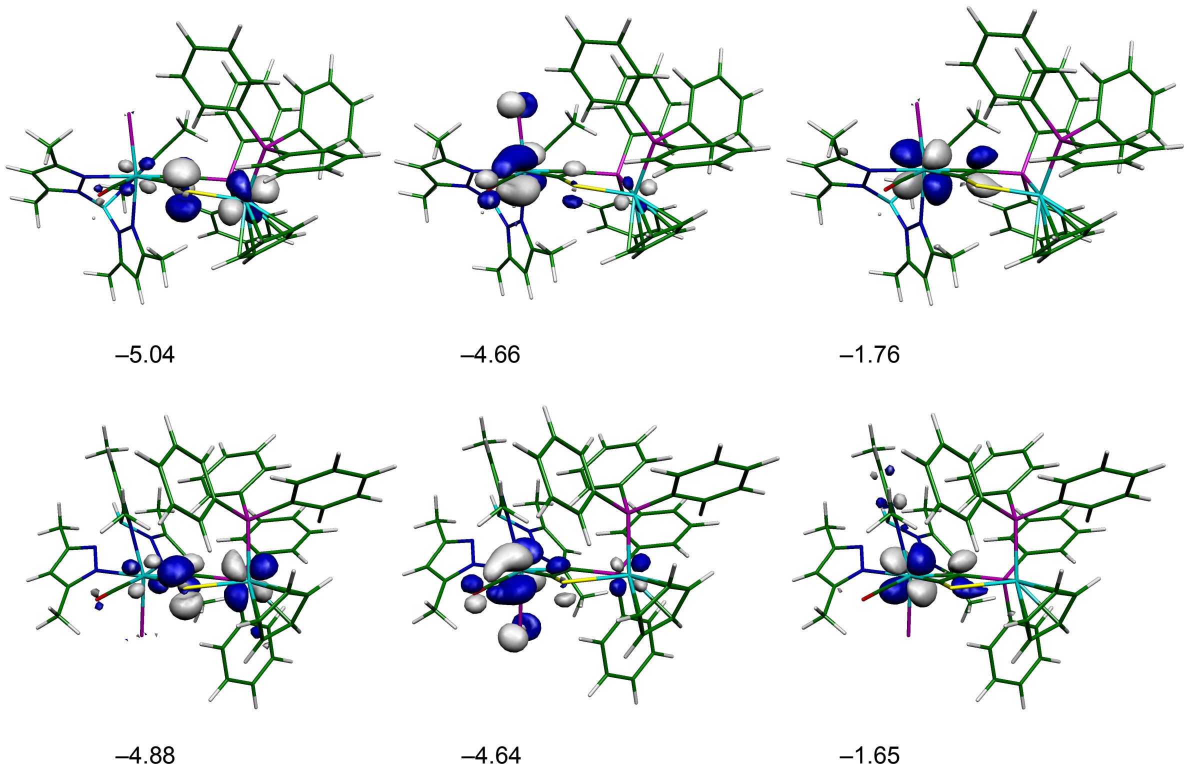

Figure S10. Kohn-Sham frontier orbitals of **3*l*** (above) and **3*u*** (below): HOMO−1 (left), HOMO (centre), LUMO (right); orbital energies given in eV.

Inspection of the frontier KS orbitals of the neutral complexes reveal a high similarity of the electronic situation with the HOMO being localized at tungsten in both cases. Hence, the *like* isomer follows qualitatively Koopmans' theorem whereas the *unlike* isomer does obviously not. This result points to specific effects of the oxidation in **3*u*** because this isomer behaves rather conspicuously. In this connection it is interesting that the calculated energy gap between the W-centred HOMO and the Ru-centred HOMO–1 is clearly smaller in the *unlike* isomer (0.24 vs. 0.38 eV).

Apparently, the metal-selective oxidation of the different diastereomers is governed by secondary interactions between the ligand spheres of both complex moieties. Geometry optimizations of both the neutral and the cationic complex species and comparison of ΔE_{tot} of the latter with that one of the cations without geometry relaxation gave an estimate of the reorganization energies λ. The higher reorganization energy of 0.25 eV for the W-based oxidation of **3*l*** (as compared with 0.20 eV for the Ru-based oxidation of **3*u***) is attributed to substantial changes of the whole coordination geometry at tungsten. Upon oxidation the bond length W–CO is increased by ~0.1 Å, whereas W–N in *trans*-position to CO and W–Br are shortened by ~0.1 Å. In addition, the Br–W–CO angle is decreased from obtuse (94.9°) to acute (86.2°). As a consequence the whole Tp' ligand changes its position with respect to the Ru complex moiety, which is reflected in the decrease of the B–W–Ru angle from 134.2° in **3*l*** to 121.7° in **3*l***$^+$ (Figure S11, Table S5). One major structural difference between **3*l*** and **3*u*** pertains to the mutual position of the Tp' ligand and the phenyl groups. In isomer **3*l*** just one phenyl group is placed in the pyrazolyl pocket, whereas two of them being mutually connected by π-stacking are directed to the Tp' ligand in **3*u*** (Figure S12). The latter could impede the structural changes necessary for relaxation

after oxidation at tungsten. The structural changes at Ru resulting from the oxidation of **3*u*** are a substantial decrease of ~0.13 Å for the Ru–S bond length accompanied by a slighter elongation of the Ru–P and Ru–Cp distances (Table S6). The dominating contraction of the Ru–S bond seems to be rather unaffected by the secondary ligand sphere interactions. The higher stability of the Ru(III) species **3*u***$^+$ is attributed to the limited geometry adaption at tungsten compared with that at ruthenium and that at tungsten in **3*l*/3*l***$^+$. In addition, in both cases, **3*l***$^+$ and ground state **3*u***$^+$, π-stacking interactions between two phenyl rings are strengthened upon oxidation (Table S5, Table S6).

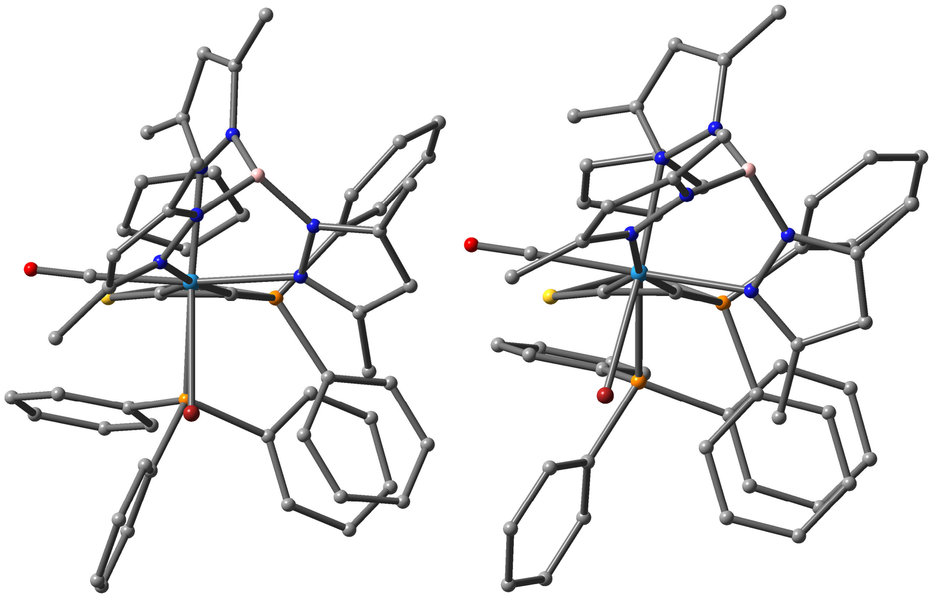

Figure S11. Optimized molecular structures of **3*l*** (left) and **3*l***$^+$ (right) in the gas phase; view along the W/Ru axis.

Table S5. Comparison of essential bond lengths [Å] and angles [°] for **3*l*** and **3*l***$^+$ obtained by single molecule geometry optimization in the gas phase, the calculated bond lengths and angles were rounded for clarity.

	**3*l***	**3*l***$^+$
W–CO	1.9641	2.0702
W–Br	2.6110	2.5153
W–C1 (alkyne)	2.0567	2.0970
W–C2 (alkyne)	2.0293	2.0122
Br–W–CO	94.93	86.16
B–W–Ru	134.24	121.72
C_{ipso}(Ph)····C_{ipso}(Ph)	3.3575	3.2905

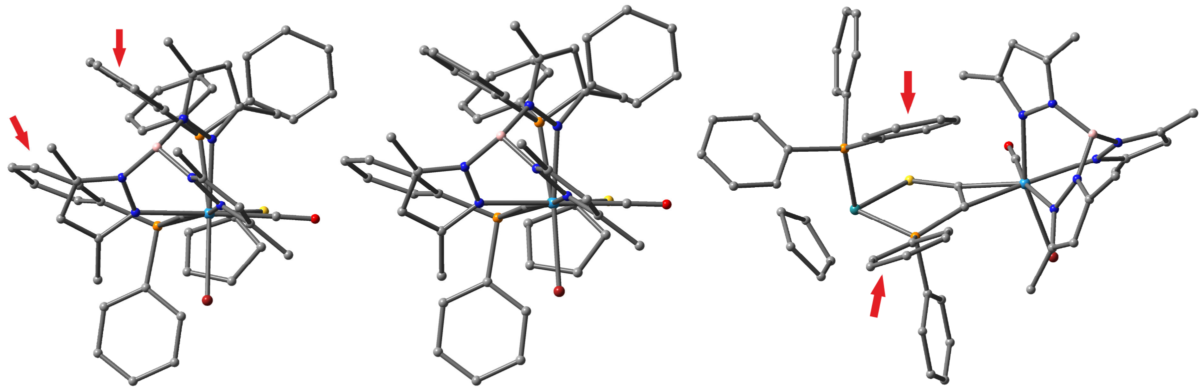

Figure S12. Optimized molecular structures of **3*u*** (left), virtual W(III)-**3*u***$^+$ (middle) and experimentally observed Ru(III)-**3*u***$^+$ in the gas phase; red arrows mark the two phenyl rings in the pyrazolyl pocket.

Table S6. Comparison of essential bond lengths [Å] and angles [°] for **3*u***, **3*u***$^+$ (Ru oxidised ground state) and **3*u***$^+$ (W oxidised local minimum state) obtained by single molecule geometry optimization in the gas phase, the calculated bond lengths and angles were rounded for clarity.

	**3*u***	**3*u***$^+$ (Ru oxidised)	**3*u***$^+$ (W oxidised)
Ru–C_5H_5 (centroid)	1.8970	1.9216	1.8959
Ru–S	2.4201	2.2924	2.3962
Ru–PPh_2 (ring)	2.2903	2.3236	2.2908
Ru–PPh_3 (terminal)	2.2846	2.3579	2.3076
B–W–Ru	131.95	132.17	132.83
C_{ipso}(Ph)····C_{ipso}(Ph)	3.2997	3.2431	3.2866
W–CO	1.9756	2.0013	2.0693
W–Br	2.5952	2.5654	2.5196

7 NMR spectra

^{1}H NMR of [Tp'W(CO)Br{(η^2-C_2H(SBn)}] (300 MHz, $CDCl_3$)

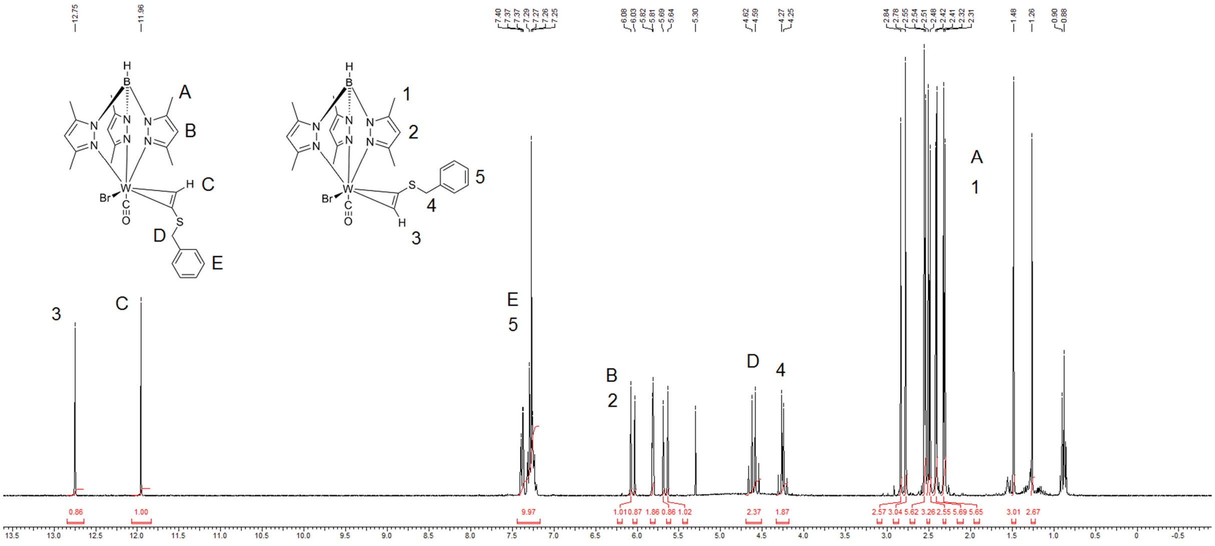

^{13}C NMR of [Tp'W(CO)Br{η^2-C_2H(SBn)}] (75 MHz, $CDCl_3$)

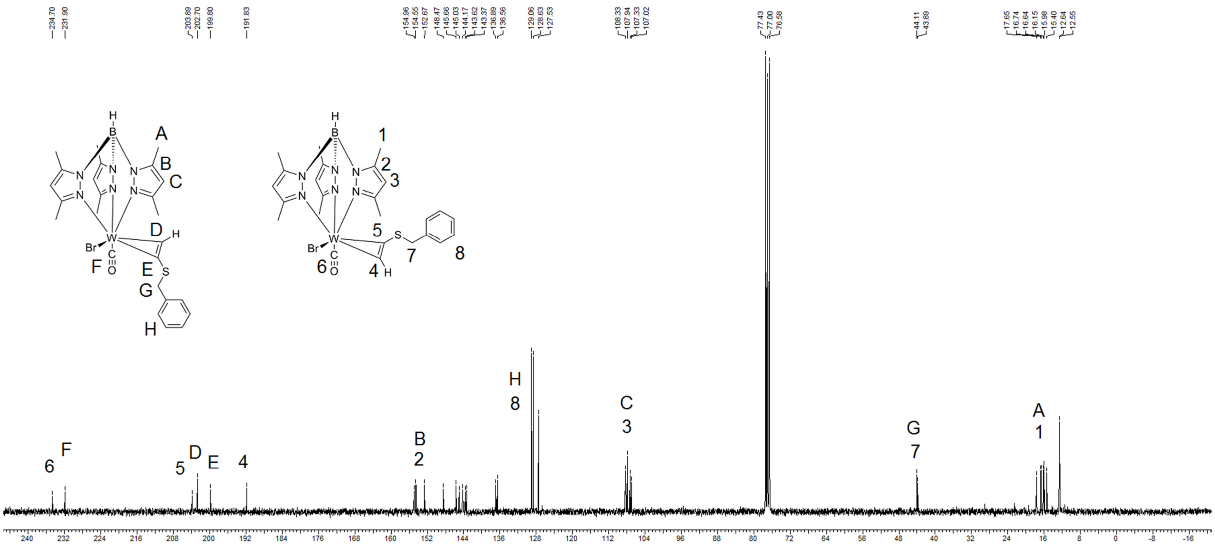

^{1}H NMR of [Tp'W(CO)Br{η^2-C_2(PPh_2)(SBn)}] **2** (250 MHz, $CDCl_3$)

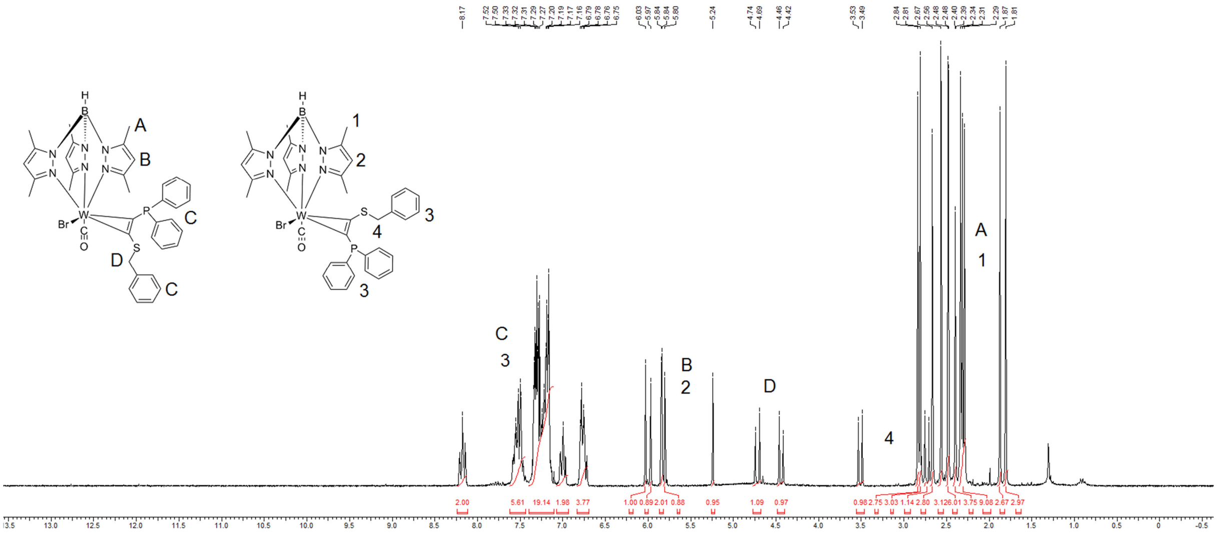

^{13}C NMR of [Tp'W(CO)Br{η^2-C_2(PPh_2)(SBn)}] **2** (63 MHz, $CDCl_3$)

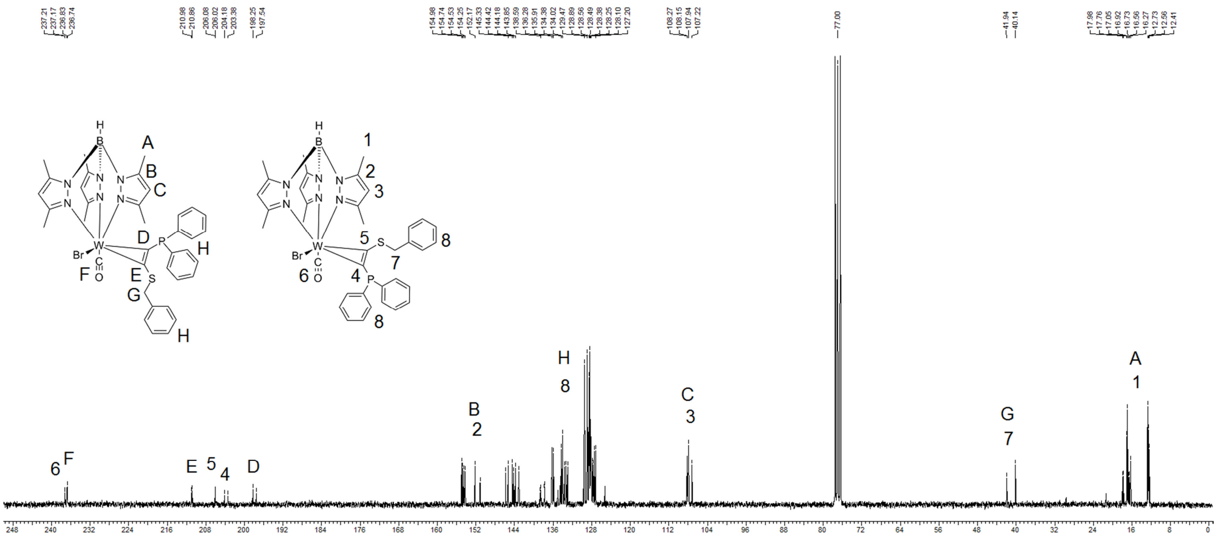

^{31}P NMR of [Tp'W(CO)Br{η^2-$C_2(PPh_2)(SBn)$}] **2** (101 MHz, $CDCl_3$)

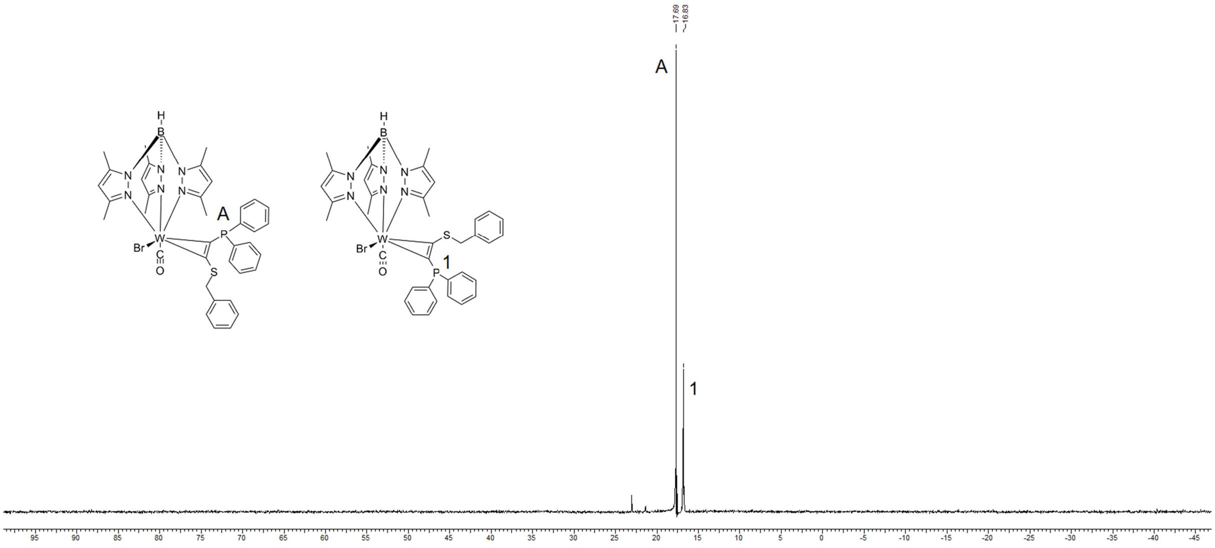

^{1}H NMR of [Tp'(CO)BrW{μ-η^2-κ^2-$C_2(PPh_2)S$}Ru(η^5-C_5H_5)(PPh_3)] **3*l*** (250 MHz, $CDCl_3$)

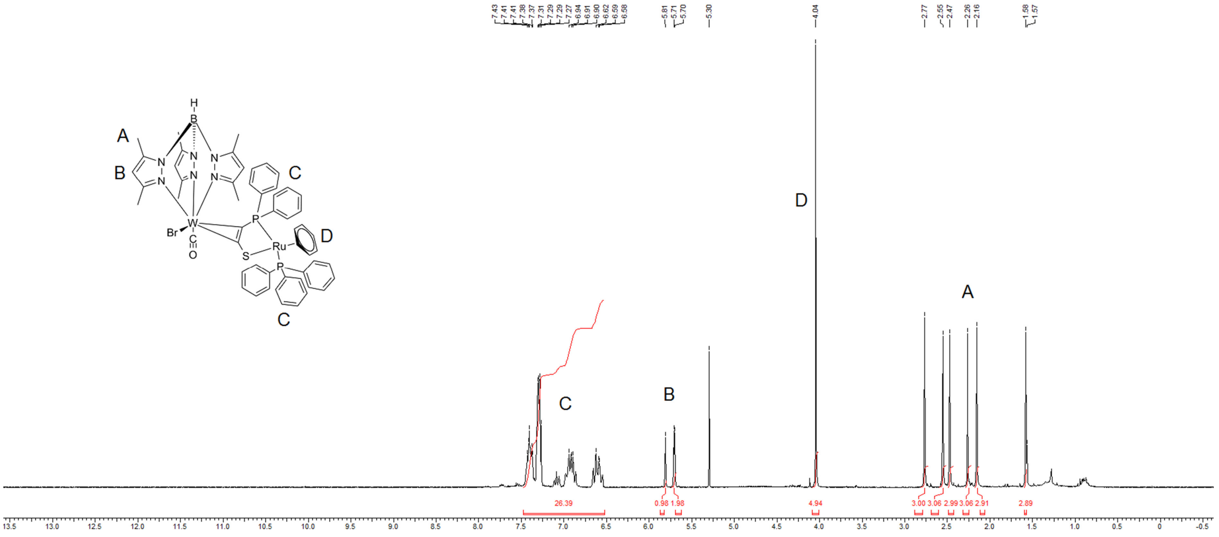

^{13}C NMR of [Tp'(CO)BrW{μ-η^2-κ^2-$C_2(PPh_2)S$}Ru(η^5-C_5H_5)(PPh_3)] **3*l*** (63 MHz, $CDCl_3$)

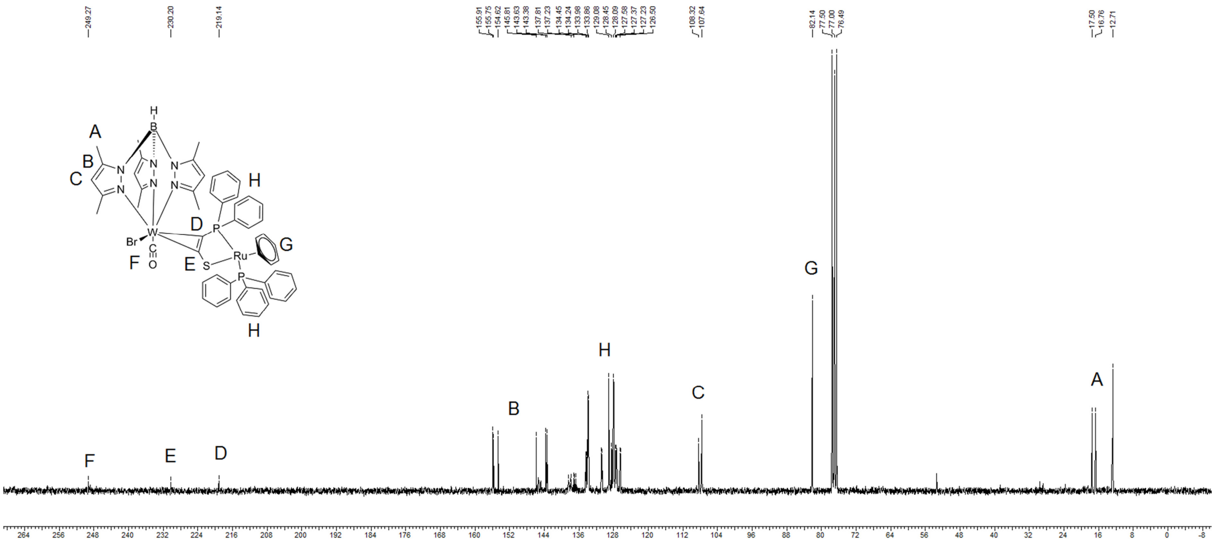

^{31}P NMR of [Tp'(CO)BrW{μ-η^2-κ^2-$C_2(PPh_2)S$}Ru(η^5-C_5H_5)(PPh_3)] **3*l*** (101 MHz, $CDCl_3$)

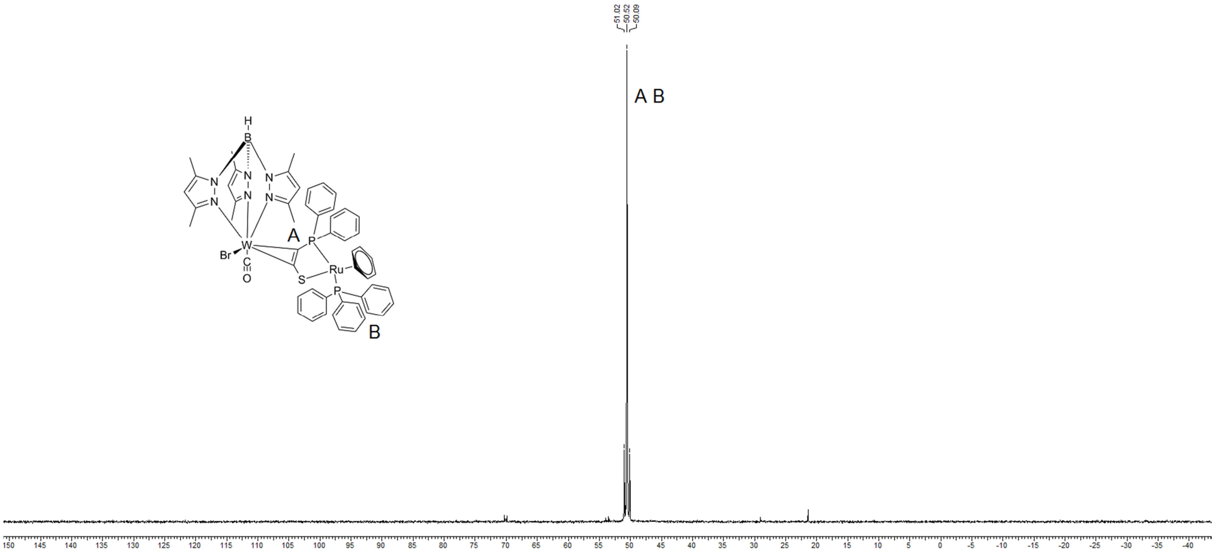

^{1}H NMR of [Tp'(CO)BrW{μ-η^2-κ^2-$C_2(PPh_2)S$}Ru(η^5-C_5H_5)(PPh_3)] ***3u*** (250 MHz, $CDCl_3$)

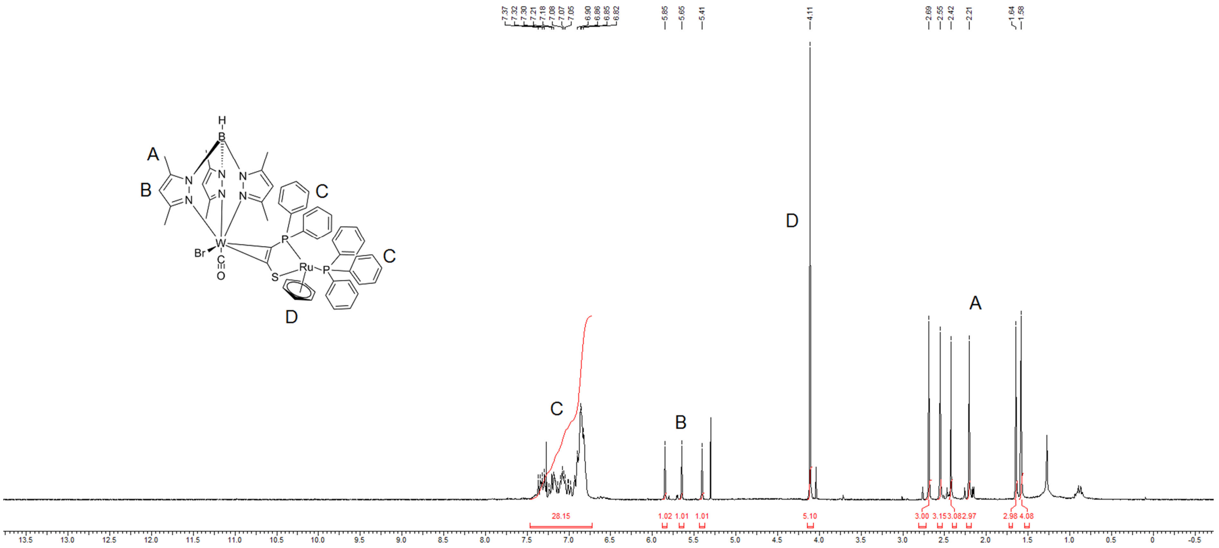

^{13}C NMR of [Tp'(CO)BrW{μ-η^2-κ^2-$C_2(PPh_2)S$}Ru(η^5-C_5H_5)(PPh_3)] ***3u*** (63 MHz, $CDCl_3$)

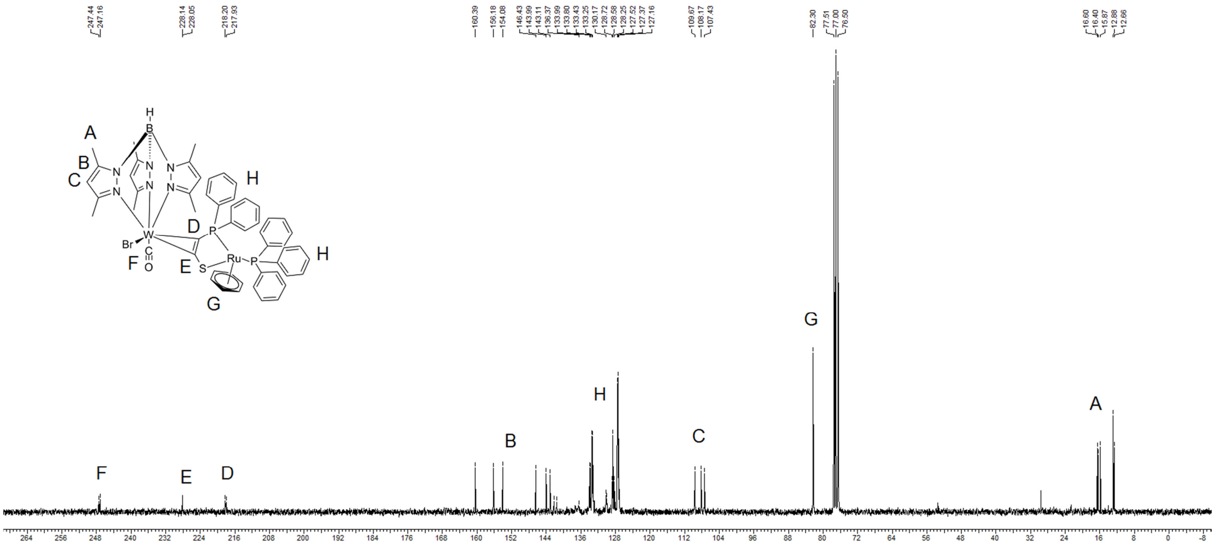

^{31}P NMR of [Tp'(CO)BrW{μ-η^2-κ^2-$C_2(PPh_2)S$}Ru(η^5-C_5H_5)(PPh_3)] ***3u*** (101 MHz, $CDCl_3$)

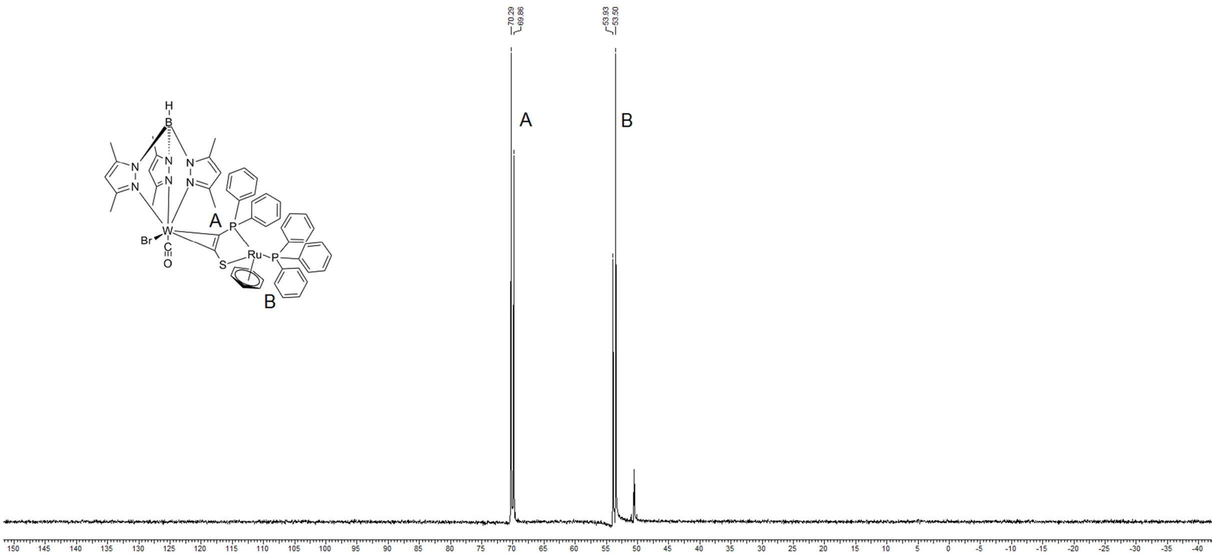

^{1}H NMR of [Tp'W(CO)I{η^2-C_2H(SBn)}] **4** (250 MHz, $CDCl_3$)

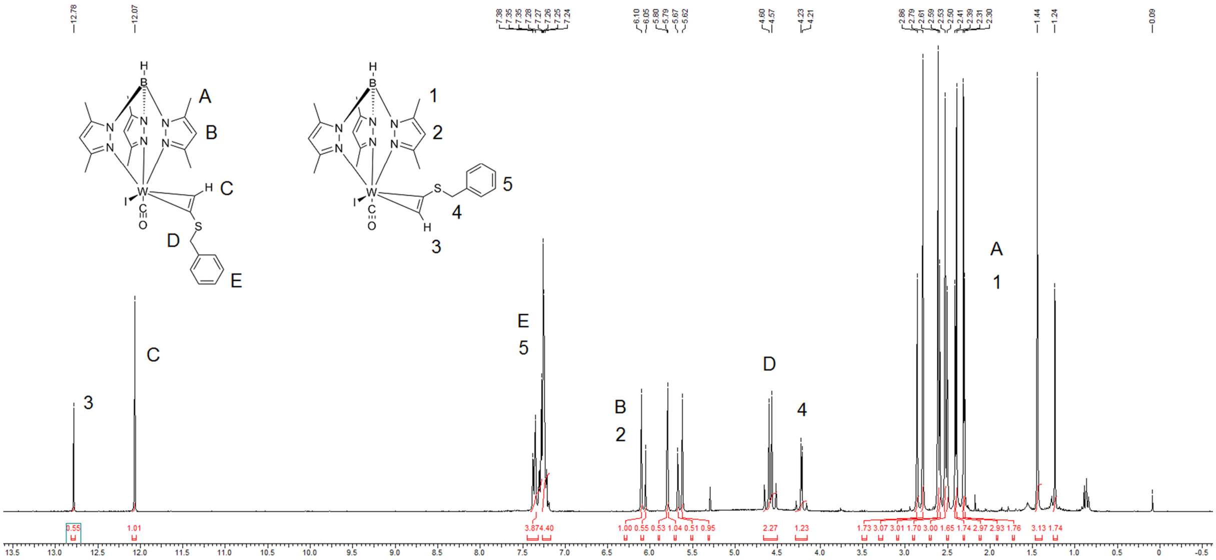

^{13}C NMR of [Tp'W(CO)I{η^2-C_2H(SBn)}] **4** (63 MHz, $CDCl_3$)

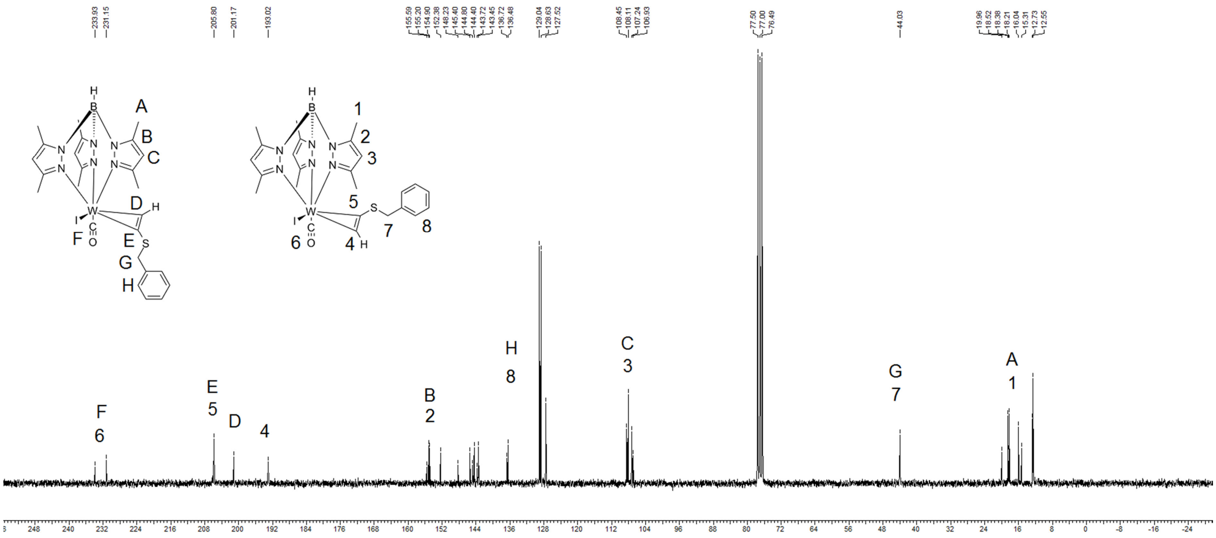

^{1}H NMR of [Tp'W(CO)(OTf){η^2-C_2H(SBn)}] (500 MHz, $CDCl_3$)

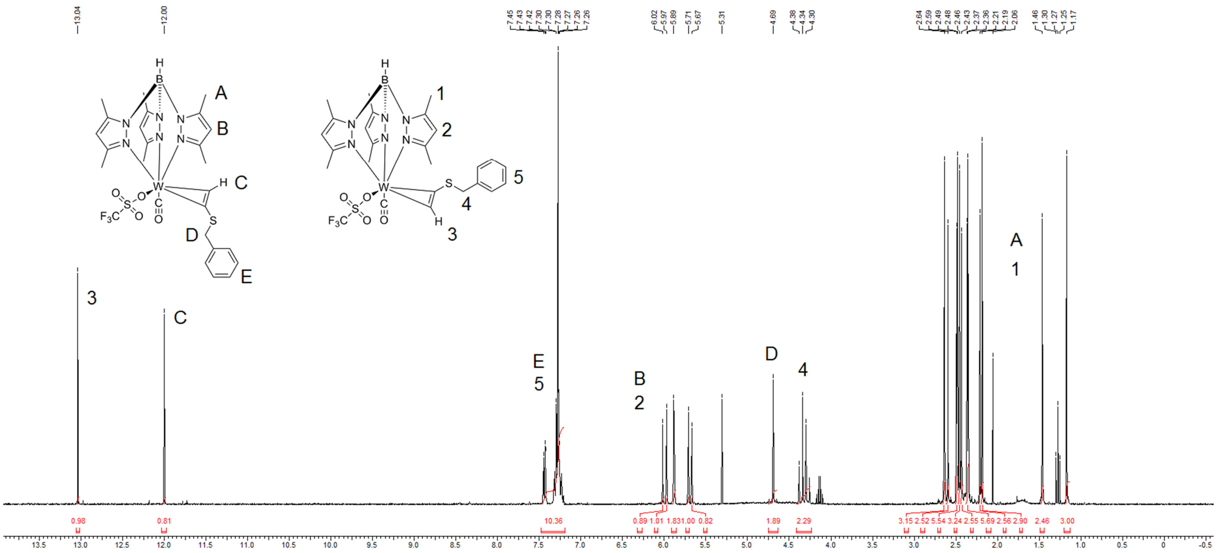

^{13}C NMR of [Tp'W(CO)(OTf){η^2-C_2H(SBn)}] (126 MHz, $CDCl_3$)

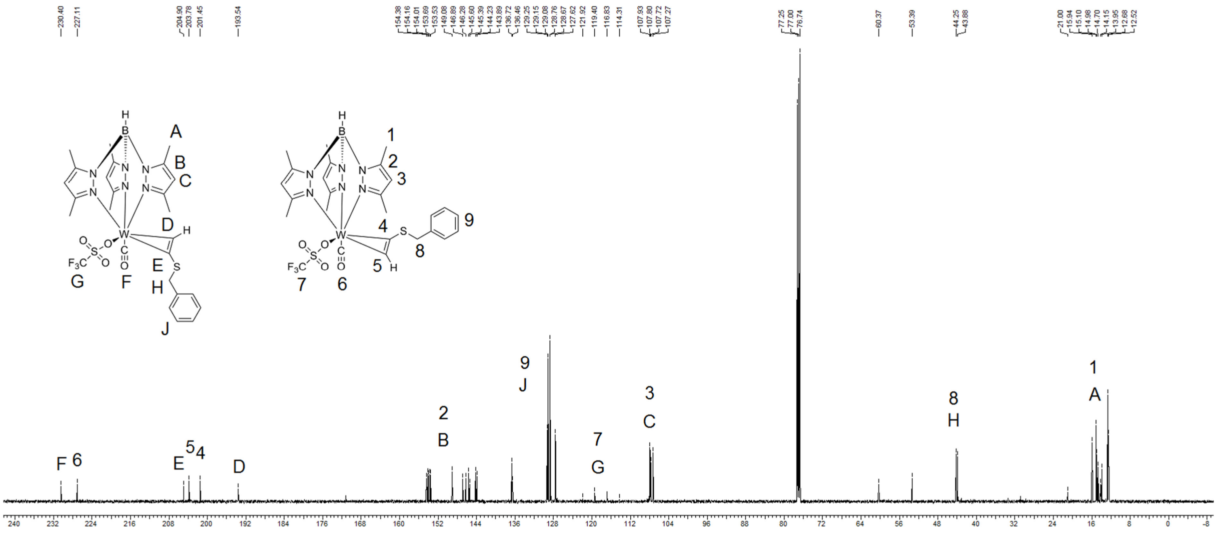

^{19}F NMR of [Tp'W(CO)(OTf){η^2-C_2H(SBn)}] (471 MHz, $CDCl_3$)

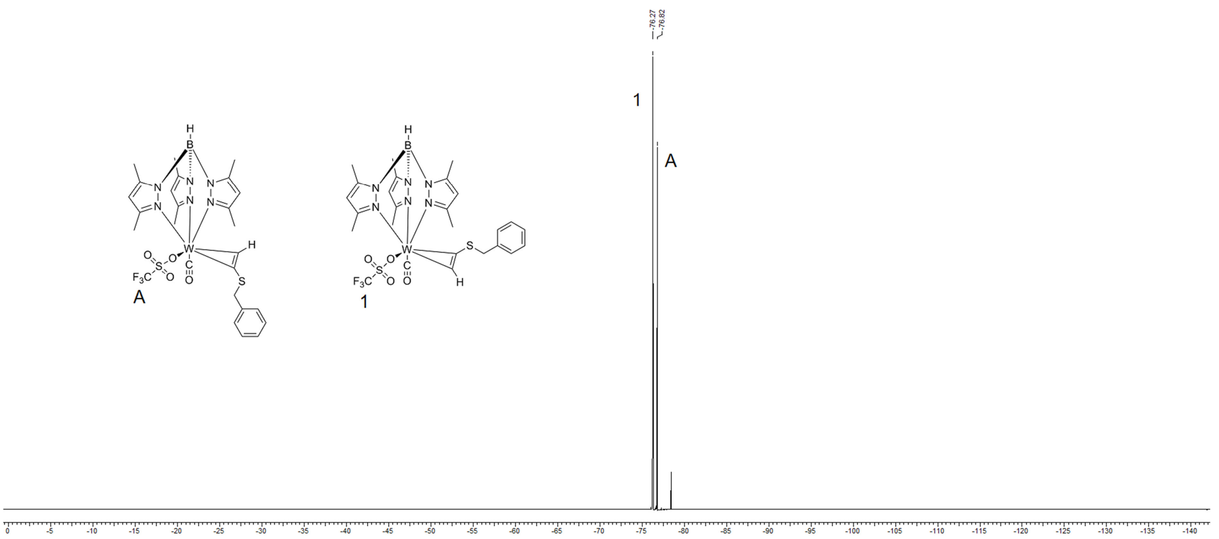

^{1}H NMR of [Tp'W(CO)(SPh){η^2-C_2H(SBn)}] **5** (250 MHz, $CDCl_3$)

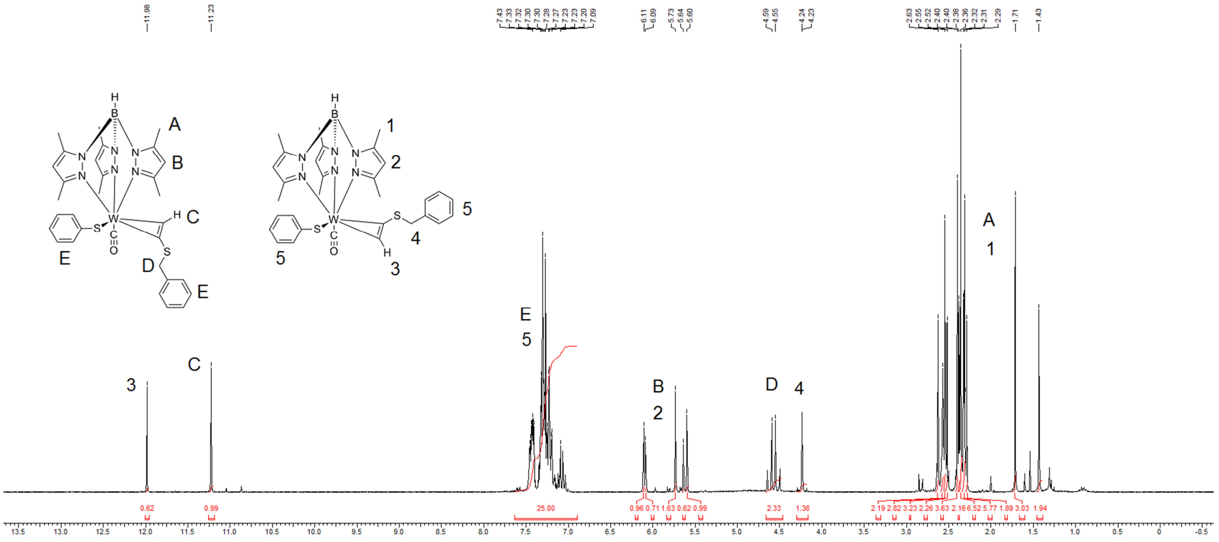

^{13}C NMR of [Tp'W(CO)(SPh){η^2-C_2H(SBn)}] **5** (63 MHz, $CDCl_3$)

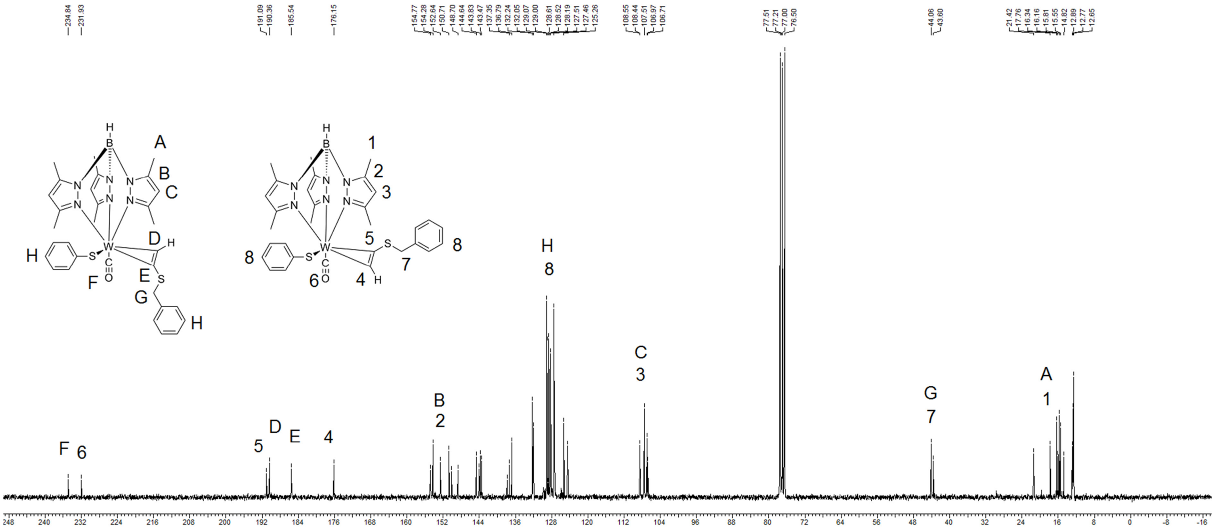

^{1}H NMR of [Tp'W(CO)(SPh){η^{2}-$C_2(PPh_2)$(SBn)}] **6** (300 MHz, $CDCl_3$)

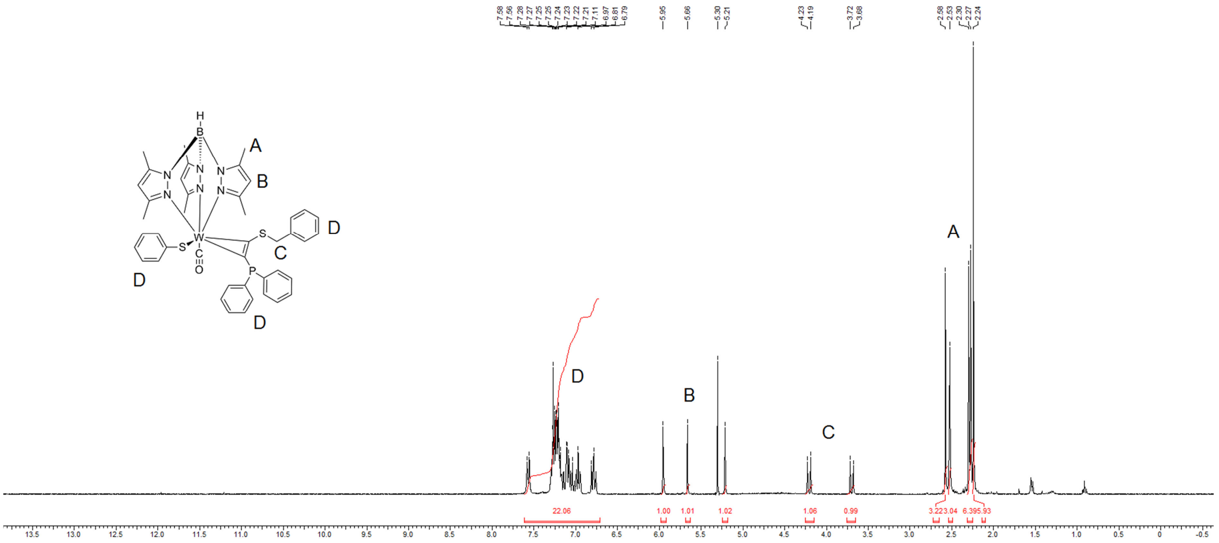

^{13}C NMR of [Tp'W(CO)(SPh){η^{2}-$C_2(PPh_2)$(SBn)}] **6** (75 MHz, $CDCl_3$)

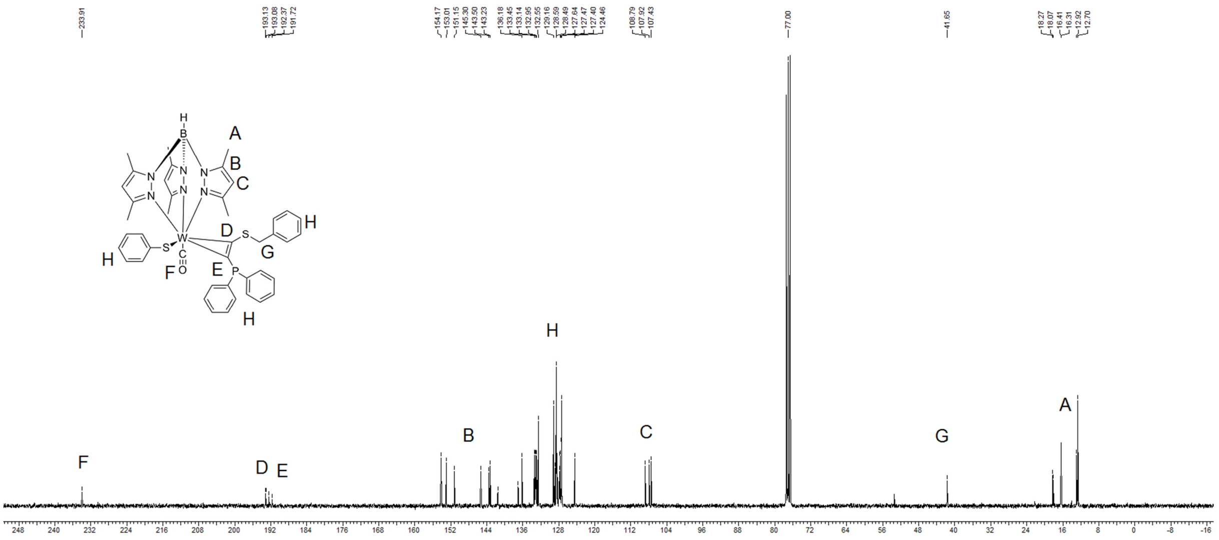

^{31}P NMR of [Tp'W(CO)(SPh){η^2-C_2(PPh_2)(SBn)}] **6** (121 MHz, $CDCl_3$)

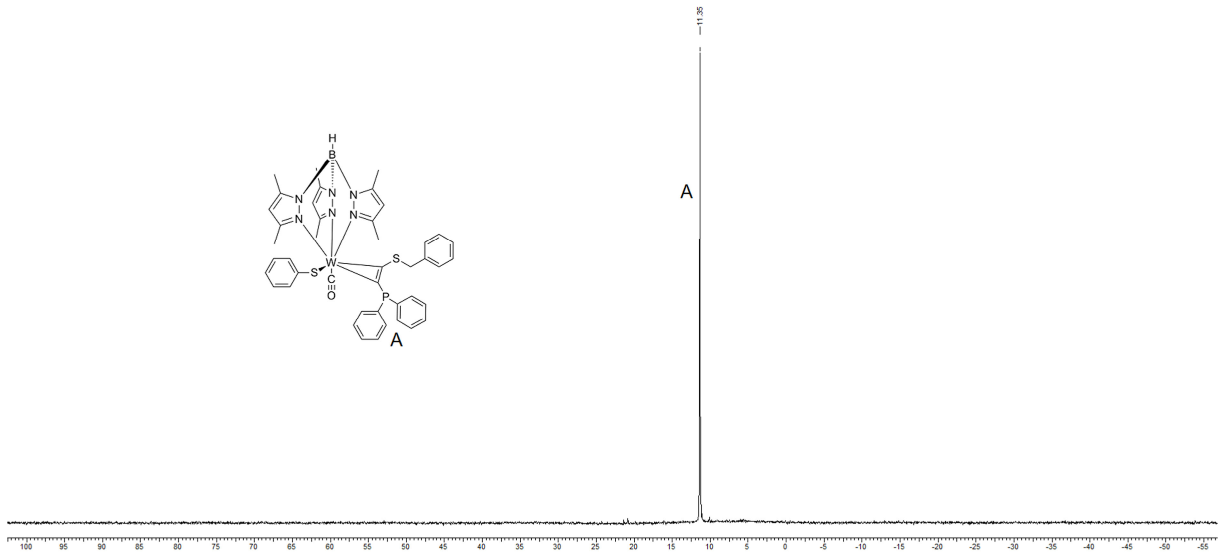

^{1}H NMR of [Tp'(CO)(SPh)W{μ-η^2-κ^2-C_2(PPh_2)S}Ru(η^5-C_5H_5)(PPh_3)] ***7u*** (500 MHz, $CDCl_3$)

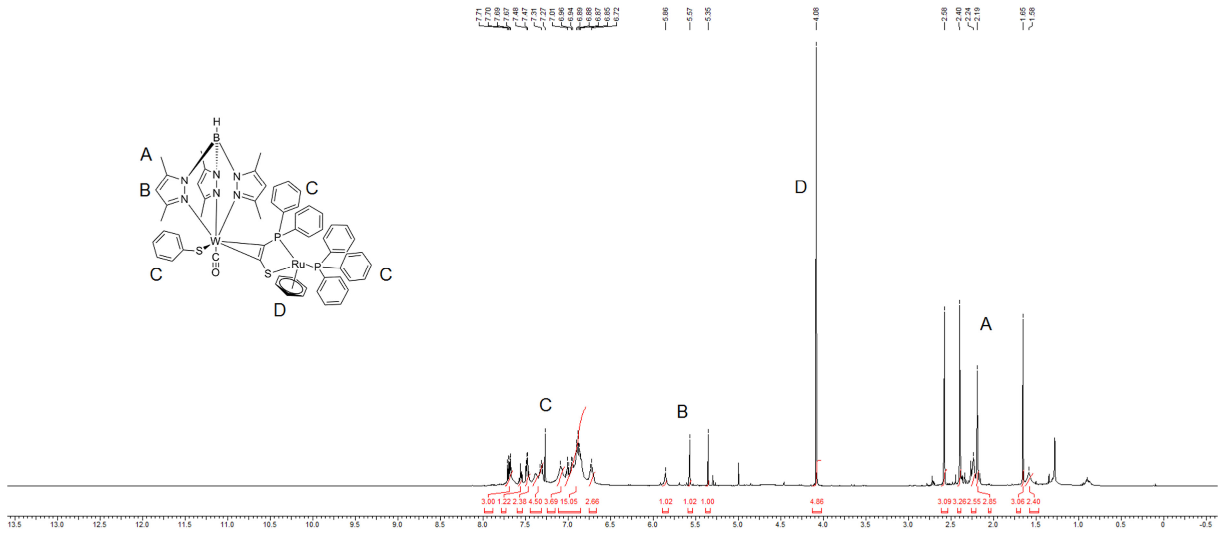

^{13}C NMR of [Tp’(CO)(SPh)W{μ-η^2-κ^2-$C_2(PPh_2)S$}Ru(η^5-C_5H_5)(PPh_3)] ***7u*** (126 MHz, $CDCl_3$)

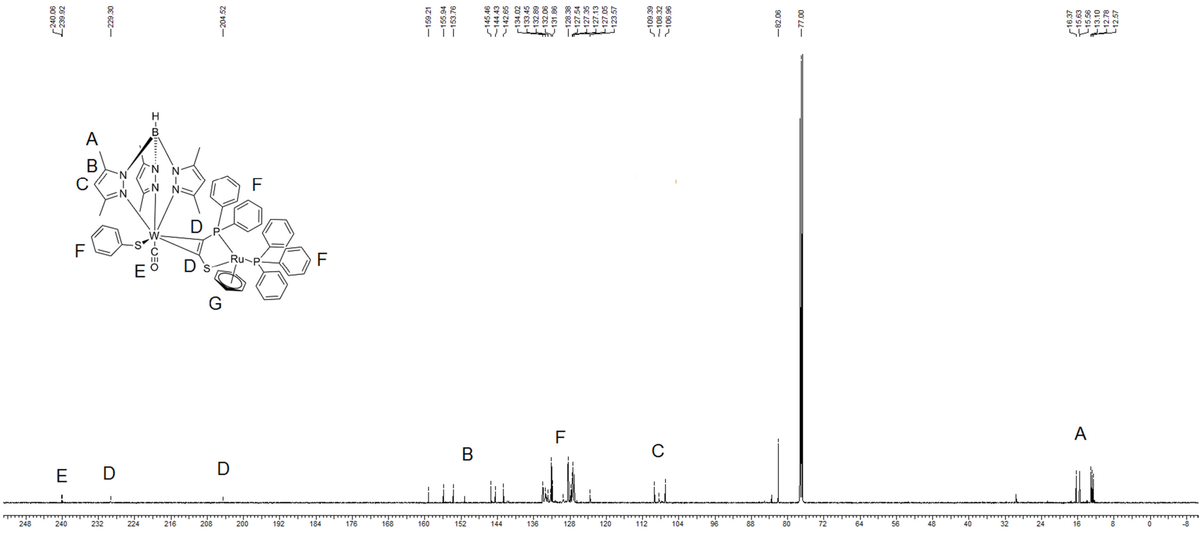

^{31}P NMR of [Tp’(CO)(SPh)W{μ-η^2-κ^2-$C_2(PPh_2)S$}Ru(η^5-C_5H_5)(PPh_3)] ***7u*** (202 MHz, $CDCl_3$)

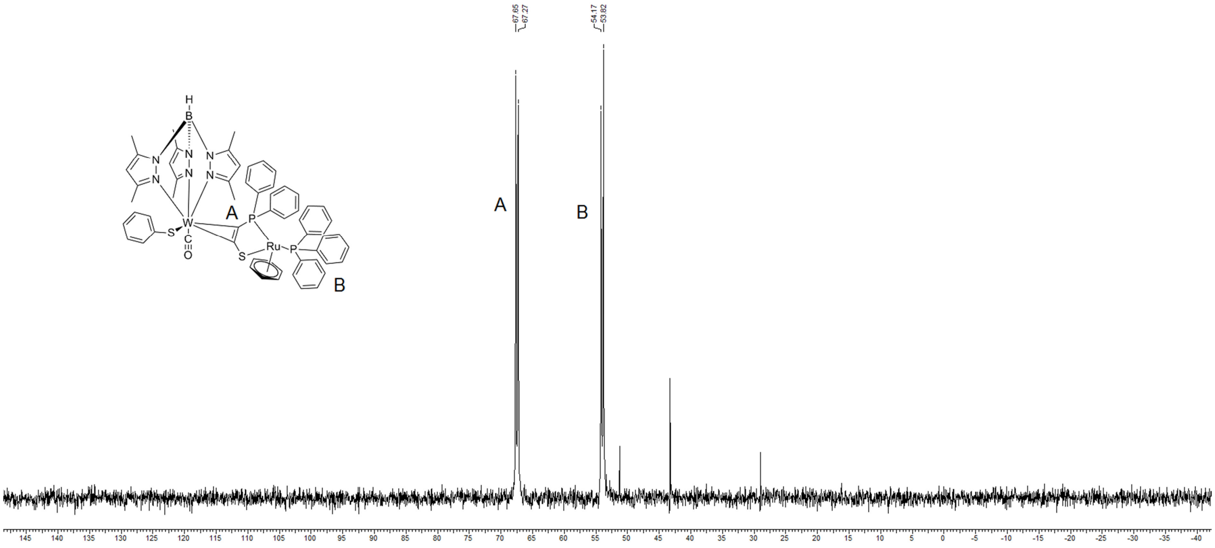

8 References

[1] C. C. Philipp, *Inorg. Chem.* **1992**, *31*, 3825.

[2] W. W. Seidel, M. J. Meel, M. Schaffrath, T. Pape, *Eur. J. Org. Chem.* **2007**, *2007*, 3526–3532.

[3] a) T. P. Gill, K. R. Mann, *Organometallics* **1982**, *1*, 485–488; b) E. Rüba, W. Simanko, K. Mauthner, K. M. Soldouzi, C. Slugovc, K. Mereiter, R. Schmid, K. Kirchner, *Organometallics* **1999**, *18*, 3843–3850.

[4] N. G. Connelly, W. E. Geiger, *Chem. Rev.* **1996**, *96*, 877–910.

[5] G. Gsaller, G. Gritzner, *Z. Phys. Chem.* **1983**, *138*, 137–155.

[6] M. Bio, G. Nkepang, Y. You, *Chem. Commun.* **2012**, *48*, 6517–6519.

[7] R. S. Cahn, C. Ingold, V. Prelog, *Angew. Chem. Int. Ed.* **1966**, *5*, 385–415.

[8] M. J. Frisch, G. W. Trucks, H. B. Schlegel, G. E. Scuseria, M. A. Robb, J. R. Cheeseman, G. Scalmani, V. Barone, B. Mennucci, G. A. Petersson, H. Nakatsuji, M. Caricato, X. Li, H. P. Hratchian, A. F. Izmaylov, J. Bloino, G. Zheng, J. L. Sonnenberg, M. Hada, M. Ehara, K. Toyota, R. Fukuda, J. Hasegawa, M. Ishida, T. Nakajima, Y. Honda, O. Kitao, H. Nakai, T. Vreven, J. A. Montgomery, Jr., J. E. Peralta, F. Ogliaro, M. Bearpark, J. J. Heyd, E. Brothers, K. N. Kudin, V. N. Staroverov, T. Keith, R. Kobayashi, J. Normand, K. Raghavachari, A. Rendell, J. C. Burant, S. S. Iyengar, J. Tomasi, M. Cossi, N. Rega, J. M. Millam, M. Klene, J. E. Knox, J. B. Cross, V. Bakken, C. Adamo, J. Jaramillo, R. Gomperts, R. E. Stratmann, O. Yazyev, A. J. Austin, R. Cammi, C. Pomelli, J. W. Ochterski, R. L. Martin, K. Morokuma, V. G. Zakrzewski, G. A. Voth, P. Salvador, J. J. Dannenberg, S. Dapprich, A. D. Daniels, O. Farkas, J. B. Foresman, J. V. Ortiz, J. Cioslowski, and D. J. Fox, *Gaussian 09, Revision E.01*, Gaussian, Inc., Wallingford CT, **2013.**

[9] a) F. Neese, *Wiley Interdiscip. Rev.: Comput. Mol. Sci.* **2012**, *2*, 73–78; b) *ORCA – An ab initio, DFT, and semi-empirical SCF-MO package*, version 4.1.1, Max Planck Institute für Kohlenforschung, Muelheim/Ruhr, Germany.

[10] a) C. Adamo, V. Barone, *J. Chem. Phys.* **1999**, *110*, 6158–6170; b) J. P. Perdew, M. Ernzerhof, K. Burke, *J. Chem. Phys.* **1996**, *105*, 9982–9985.

[11] a) D. Andrae, U. Huermann, M. Dolg, H. Stoll, H. Preu, *Theor. Chim. Acta* **1990**, *77*, 123–141; b) A. Bergner, M. Dolg, W. Küchle, H. Stoll, H. Preuß, *Mol. Phys.* **1993**, *80*, 1431–1441; c) J. M. L. Martin, A. Sundermann, *J. Chem. Phys.* **2001**, *114*, 3408–3420.

[12] F. Weigend, R. Ahlrichs, *Phys. Chem. Chem. Phys.* **2005**, *7*, 3297–3305.

[13] a) S. Grimme, S. Ehrlich, L. Goerigk, *J. Comput. Chem.* **2011**, *32*, 1456–1465; b) S. Grimme, J. Antony, S. Ehrlich, H. Krieg, *J. Chem. Phys.* **2010**, *132*, 154104.

9 Cartesian coordinates of calculated complexes

Table S7. Cartesian coordinates of the geometry optimized structures of **3*l*** (left) and **3*u*** (right) in the gas phase; b3lyp, def2-TZVP/ECP(W,Ru).

W	-2.247956	-0.817092	-0.236441	Ru	2.894504	1.002100	-0.994648
N	-3.289168	0.385412	1.445852	C	3.852529	3.029739	-0.869649
N	-4.358475	-1.693292	-0.409884	C	4.858038	2.052651	-0.639517
N	-3.189934	0.727050	-1.520113	C	3.337620	2.856749	-2.180721
C	-0.495153	0.169683	0.031623	C	4.040702	1.757351	-2.772281
C	-0.331519	-0.711674	-0.975557	C	4.976558	1.269242	-1.832748
C	-1.974927	-2.003400	-1.777780	P	1.189446	1.515542	0.445668
N	-4.547633	0.882249	1.223881	P	3.308886	-1.064241	-0.112736
C	-2.953809	0.748386	2.694174	S	1.108313	0.242443	-2.440117
N	-5.445678	-0.870872	-0.312364	W	-2.186640	0.189313	-0.774230
C	-4.822428	-2.937260	-0.632068	C	-2.046630	-0.237552	-2.698044
N	-4.433466	1.219314	-1.237655	C	-0.339789	0.751181	-0.144740
C	-2.736097	1.399172	-2.592976	C	-0.242904	0.354118	-1.429927
P	0.935747	1.230949	0.335855	N	-2.118259	-1.960821	-0.260981
S	0.991622	-1.022042	-1.977071	N	-2.914361	0.622991	1.348943
O	-1.818142	-2.649448	-2.723244	N	-4.373630	-0.499207	-0.947901
B	-5.272234	0.635156	-0.094157	C	-2.803239	1.665440	2.184734
C	-4.994746	1.541705	2.309803	N	-3.545220	-0.375215	2.040311
C	-1.653680	0.452222	3.335362	C	-3.823282	0.028062	3.297586
C	-4.000576	1.478909	3.265936	B	-4.064765	-1.605851	1.306161
C	-6.579881	-1.579724	-0.459391	C	-5.329491	-0.372711	-1.882440
C	-3.979519	-4.146603	-0.841361	N	-4.930144	-1.161521	0.112254
C	-6.219323	-2.900067	-0.660466	C	-6.223797	-1.435503	-0.141839
C	-4.762021	2.186574	-2.115698	C	-1.467498	-3.009418	-0.801446
C	-1.407381	1.179908	-3.209864	N	-2.911410	-2.456320	0.744342
C	-3.697861	2.333040	-2.986820	C	-2.758746	-3.791944	0.836331
Ru	2.424913	0.823774	-1.388741	O	-1.938233	-0.457364	-3.825618
C	1.431639	0.911056	2.071876	C	1.362147	1.294130	2.241478
C	0.204772	2.910457	0.422856	C	0.780707	3.301360	0.301059
C	-6.336260	2.186027	2.390847	C	2.389591	-1.710090	1.342951
C	-7.941077	-0.976711	-0.397775	C	5.060736	-1.214880	0.428067
P	3.866957	-0.480856	-0.171422	C	3.149721	-2.497792	-1.255965
C	1.963654	2.688696	-2.581014	C	-2.175724	2.968406	1.850178
C	2.123125	1.614205	-3.480957	C	-3.355513	1.320349	3.426494
C	3.456500	1.115070	-3.370804	C	-4.517731	-0.826949	4.301353
C	4.122138	1.909082	-2.395491	C	-5.130566	0.247385	-3.220802
C	3.210813	2.872927	-1.895163	C	-6.512600	-0.943910	-1.402335
C	2.047756	1.895476	2.845731	C	-7.117214	-2.142647	0.818453
C	1.262845	-0.360779	2.615955	C	-0.539883	-2.940850	-1.961237
C	-1.180310	3.081141	0.428239	C	-1.831113	-4.171364	-0.116402
C	1.021491	4.043211	0.456987	C	-3.493171	-4.638195	1.817837
C	4.644984	0.349992	1.276442	C	2.577698	1.641640	2.834465
C	3.525261	-2.178808	0.445777	C	0.326566	0.819329	3.038878

C	5.333241	-0.863821	-1.220164	C	2.746496	1.537427	4.207276
C	2.475238	1.613323	4.134878	C	1.702773	1.070896	5.000507
C	1.697655	-0.645514	3.902016	C	0.497773	0.708509	4.412664
C	-1.733378	4.355277	0.465947	C	0.234468	3.750606	-0.903609
C	4.962083	1.705958	1.157286	C	1.034448	4.221983	1.316451
C	4.967581	-0.306003	2.461724	C	-0.061513	5.093521	-1.081605
C	4.589223	-3.040778	0.739756	C	0.199637	6.009185	-0.068035
C	2.226241	-2.642267	0.631477	C	0.750490	5.569983	1.129093
C	6.611103	-0.365683	-0.983025	C	5.524509	-0.394483	1.461237
C	5.123378	-1.686505	-2.331151	C	5.962560	-2.077008	-0.192940
C	2.303195	0.341257	4.667616	C	7.296328	-2.113579	0.203644
C	5.621008	2.377257	2.175913	C	7.743622	-1.300337	1.235150
C	5.613610	0.370343	3.490505	C	6.849143	-0.442133	1.868102
C	4.352278	-4.315435	1.232208	C	3.284303	-2.288168	-2.627462
C	1.984050	-3.919717	1.122284	C	2.929868	-3.795586	-0.791037
C	7.659438	-0.671700	-1.847833	C	2.840502	-4.857950	-1.680820
C	6.168361	-1.997384	-3.186528	C	2.970047	-4.637164	-3.047651
C	5.954302	1.707657	3.348283	C	3.196064	-3.349709	-3.518797
C	3.046829	-4.757393	1.428735	C	2.972391	-2.018819	2.571093
C	7.442193	-1.485629	-2.950010	C	1.017033	-1.904947	1.187643
C	-6.062425	2.913116	-2.089495	C	2.195390	-2.498581	3.619539
C	-0.913279	5.475374	0.505349	C	0.831113	-2.688906	3.451066
C	0.468691	5.315274	0.504296	C	0.243146	-2.391267	2.228281
H	-6.337458	1.161647	-0.076243	H	3.523957	3.775809	-0.165694
H	-1.769052	0.440894	4.418967	H	5.468147	1.955935	0.240211
H	-1.269890	-0.508176	3.015257	H	5.658362	0.448827	-1.981202
H	-0.919349	1.217887	3.088797	H	3.878535	1.370334	-3.764814
H	-4.023404	1.899428	4.256534	H	2.563946	3.443201	-2.643918
H	-4.559393	-5.034241	-0.589606	H	-2.940991	3.737261	1.733890
H	-3.672178	-4.229676	-1.884843	H	-4.570582	1.177190	-3.151367
H	-3.084551	-4.127163	-0.225817	H	-4.713253	-2.270011	2.048978
H	-6.880298	-3.736722	-0.807869	H	-1.504488	3.274495	2.653206
H	-1.328121	1.773070	-4.120189	H	-1.621647	2.923150	0.924730
H	-0.607094	1.476499	-2.533740	H	-3.400314	1.942658	4.303847
H	-1.237575	0.133114	-3.455176	H	-4.551307	-0.308244	5.258234
H	-3.623362	3.021888	-3.810416	H	-5.542312	-1.051915	4.000303
H	1.069894	3.269826	-2.431803	H	-4.004509	-1.779027	4.444776
H	1.355314	1.203937	-4.113931	H	-6.101242	0.452190	-3.670857
H	3.890500	0.313722	-3.942076	H	-4.585366	-0.423992	-3.885668
H	5.150113	1.795003	-2.096751	H	-7.458241	-0.989362	-1.914544
H	3.440423	3.655442	-1.194219	H	-8.120405	-2.207455	0.400345
H	-6.449247	2.663156	3.363059	H	-6.767626	-3.155419	1.025267
H	-6.468251	2.946338	1.619432	H	-7.177740	-1.616016	1.772063
H	-7.141241	1.458971	2.272582	H	-0.016711	-3.889914	-2.060791
H	-8.688976	-1.755722	-0.536187	H	-1.088900	-2.752460	-2.884143
H	-8.123909	-0.494024	0.563407	H	0.203453	-2.154912	-1.873174
H	-8.083751	-0.223809	-1.174430	H	-1.456284	-5.163765	-0.297571
H	2.189911	2.890655	2.452322	H	-3.102018	-5.653774	1.784559

H	0.741890	-1.122453	2.059821	H	-3.384984	-4.260086	2.835097
H	-1.825879	2.217839	0.400899	H	-4.560985	-4.676422	1.596063
H	2.095678	3.929049	0.440891	H	3.388216	1.993384	2.208657
H	2.954574	2.387452	4.719396	H	-0.605306	0.524802	2.583158
H	1.545150	-1.638362	4.304763	H	3.691437	1.812869	4.658271
H	-2.810127	4.465516	0.465203	H	1.834295	0.980855	6.071176
H	4.677753	2.234201	0.259727	H	-0.314582	0.330621	5.019860
H	4.703190	-1.343584	2.595378	H	0.018228	3.048184	-1.695208
H	5.608126	-2.715852	0.579995	H	1.438607	3.891052	2.262230
H	1.391490	-2.022257	0.367346	H	-0.511565	5.419749	-2.009856
H	6.797454	0.259610	-0.121478	H	-0.033289	7.056872	-0.208060
H	4.134779	-2.085508	-2.520547	H	0.950813	6.274436	1.926565
H	2.641911	0.122211	5.672052	H	4.838450	0.282704	1.950375
H	5.866470	3.425378	2.058982	H	5.628510	-2.723362	-0.991633
H	5.842636	-0.153611	4.409561	H	7.983304	-2.784702	-0.295954
H	5.187581	-4.966568	1.456761	H	8.779989	-1.332262	1.545386
H	0.960304	-4.244580	1.253656	H	7.186372	0.194198	2.676610
H	8.647434	-0.274899	-1.651350	H	3.444414	-1.284587	-2.994059
H	5.991043	-2.640309	-4.039297	H	2.821057	-3.977322	0.268806
H	6.460807	2.229949	4.149750	H	2.666122	-5.858624	-1.305998
H	2.862722	-5.754697	1.807688	H	2.890641	-5.463960	-3.741873
H	8.257648	-1.725398	-3.620050	H	3.290317	-3.167816	-4.581501
H	-6.078032	3.655336	-2.885995	H	4.033909	-1.894929	2.719250
H	-6.905338	2.236088	-2.236368	H	0.547621	-1.663533	0.249780
H	-6.218734	3.426656	-1.139588	H	2.663250	-2.721615	4.569918
H	-1.344893	6.467614	0.533787	H	0.227257	-3.056147	4.271098
H	1.116302	6.182373	0.533678	H	-0.819511	-2.515264	2.087836
Br	-1.818921	-2.670451	1.551825	Br	-3.137854	2.527827	-1.375694

Table S8. Cartesian coordinates of the geometry optimized structures of **3*l***$^{+}$ in the gas phase; b3lyp (left) and PBE0 (right), def2-TZVP/ECP(W,Ru).

W	-2.415474	-0.989173	-0.222767	W	-2.240018	-0.707304	-0.115631
N	-3.081602	0.269406	1.478196	N	-3.313857	0.475179	1.433799
N	-4.639184	-1.331943	-0.360845	N	-4.275729	-1.716555	-0.316104
N	-3.065905	0.581251	-1.616298	N	-3.168037	0.746694	-1.446341
C	-0.621350	-0.110532	0.018539	C	-0.503160	0.308697	0.120924
C	-0.414066	-1.120187	-0.834889	C	-0.320073	-0.604367	-0.858801
C	-2.231375	-2.333044	-1.786729	C	-1.859086	-2.037654	-1.595056
N	-4.045329	1.220300	1.260155	N	-4.610130	0.830820	1.209842
C	-2.636859	0.441400	2.738919	C	-3.018672	0.869175	2.687505
N	-5.473924	-0.264468	-0.146218	N	-5.395241	-0.952685	-0.336289
C	-5.423465	-2.406395	-0.591102	C	-4.667748	-2.986744	-0.542152
N	-4.054874	1.451328	-1.239725	N	-4.451473	1.144739	-1.244500
C	-2.624648	0.981395	-2.828591	C	-2.710670	1.429822	-2.519672
P	0.834620	0.931647	0.348760	P	0.9690456	1.3685368	0.3709836
S	0.946805	-1.563039	-1.703527	S	0.9691455	-0.9322420	-1.8565797
O	-2.058616	-3.068732	-2.632558	O	-1.608796	-2.761051	-2.433628
B	-4.911393	1.157580	0.005842	B	-5.297423	0.548413	-0.118388
C	-4.186440	1.994876	2.351657	C	-5.128116	1.428488	2.291845
C	-1.672891	-0.447795	3.430419	C	-1.708003	0.695203	3.338180
C	-3.300833	1.528992	3.307044	C	-4.137022	1.468268	3.252751
C	-6.757118	-0.660926	-0.221499	C	-6.478587	-1.702662	-0.577745
C	-4.929859	-3.769020	-0.929972	C	-3.787952	-4.177685	-0.652898
C	-6.758598	-2.018644	-0.497813	C	-6.050601	-3.009912	-0.698188
C	-4.217062	2.397100	-2.179363	C	-4.810638	2.050016	-2.161924
C	-1.615616	0.251604	-3.637529	C	-1.341238	1.326680	-3.057894
C	-3.316625	2.129317	-3.201371	C	-3.722172	2.253071	-2.992866
Ru	2.242945	0.487554	-1.427285	Ru	2.4108008	0.8585019	-1.3555433
C	1.243520	0.433598	2.059491	C	1.4712624	1.0709459	2.1002781
C	0.187745	2.626463	0.500700	C	0.2246099	3.0325871	0.4393355
C	-5.148115	3.128184	2.434020	C	-6.526151	1.919400	2.378756
C	-7.913262	0.257389	-0.029749	C	-7.848988	-1.147732	-0.705975
P	4.041463	-0.305290	-0.182159	P	3.8801017	-0.4653425	-0.1357041
C	1.307979	1.973950	-2.823872	C	2.0243429	2.7030708	-2.5247163
C	1.836573	0.917565	-3.606581	C	2.1069906	1.6116402	-3.4150149
C	3.242907	0.857388	-3.421479	C	3.4110592	1.0425695	-3.3226308
C	3.592713	1.903368	-2.513537	C	4.1366985	1.8107245	-2.3737774
C	2.410166	2.584237	-2.137338	C	3.2910168	2.8283263	-1.8670922
C	1.331391	1.318736	3.130562	C	2.1648092	2.0497000	2.8079021
C	1.486820	-0.924131	2.276544	C	1.2494479	-0.1652944	2.6998174
C	-1.151324	2.914156	0.245522	C	-1.158184	3.172661	0.527920
C	1.056300	3.672978	0.824890	C	1.0148764	4.1813040	0.4097016
C	4.419296	0.765431	1.264771	C	4.7036838	0.3416126	1.2836927
C	4.279097	-2.012736	0.458133	C	3.4985605	-2.1514719	0.4627830
C	5.587054	-0.206473	-1.178060	C	5.3082703	-0.8794184	-1.2104676
C	1.666560	0.849087	4.395048	C	2.6128443	1.7984127	4.0940109

C	1.812382	-1.391879	3.538917	C	1.6974323	-0.4154105	3.9857960
C	-1.616535	4.221091	0.316856	C	-1.738975	4.428305	0.598962
C	4.408470	2.150666	1.072008	C	5.1052049	1.6700914	1.1411984
C	4.698746	0.275877	2.538042	C	5.0132106	-0.3280974	2.4628074
C	5.527715	-2.374728	0.979743	C	4.5374062	-3.0713357	0.6247800
C	3.280269	-2.978988	0.391569	C	2.1985425	-2.5639590	0.7277790
C	6.624303	0.685025	-0.918103	C	6.6155139	-0.5127601	-0.9131540
C	5.712171	-1.096069	-2.249172	C	5.0613753	-1.6192285	-2.3677111
C	1.903949	-0.503202	4.604492	C	2.3824137	0.5645170	4.6861131
C	4.692758	3.019062	2.115159	C	5.8211047	2.3069908	2.1394505
C	4.967825	1.146112	3.588160	C	5.7213619	0.3146557	3.4691164
C	5.750202	-3.656532	1.457147	C	4.2738474	-4.3674732	1.0324316
C	3.504124	-4.268125	0.863597	C	1.9319743	-3.8605948	1.1410630
C	7.758768	0.700091	-1.725785	C	7.6550957	-0.8505938	-1.7705895
C	6.843920	-1.084339	-3.048831	C	6.0990611	-1.9668435	-3.2143329
C	4.973403	2.517726	3.381713	C	6.1301672	1.6287976	3.3109804
C	4.735256	-4.607882	1.405418	C	2.9683660	-4.7682431	1.2829773
C	7.871095	-0.179853	-2.792254	C	7.4003734	-1.5741647	-2.9229887
C	-5.213979	3.497300	-2.073050	C	-6.155884	2.672287	-2.216134
C	-0.751836	5.255298	0.649215	C	-0.945280	5.564549	0.580937
C	0.587153	4.976314	0.906014	C	0.4341305	5.4372532	0.4879183
H	-5.793433	1.947312	0.076490	H	-6.384004	1.036770	-0.128559
H	-2.206549	-1.272913	3.906297	H	-1.723903	1.165086	4.320591
H	-0.947135	-0.877527	2.755061	H	-1.456676	-0.358671	3.457246
H	-1.144474	0.107093	4.202085	H	-0.914386	1.156647	2.756342
H	-3.159830	1.915954	4.301146	H	-4.211719	1.881618	4.245027
H	-5.748889	-4.479989	-0.839370	H	-4.313900	-5.044036	-0.250896
H	-4.572281	-3.812881	-1.960256	H	-3.555820	-4.393355	-1.698734
H	-4.122628	-4.088544	-0.275715	H	-2.854921	-4.067136	-0.108068
H	-7.620091	-2.651923	-0.619227	H	-6.660057	-3.878284	-0.887403
H	-1.286027	0.882721	-4.460642	H	-1.252693	1.977627	-3.926806
H	-0.748911	-0.041087	-3.055680	H	-0.600785	1.638018	-2.318038
H	-2.046093	-0.653714	-4.070100	H	-1.082948	0.309333	-3.357146
H	-3.187674	2.688735	-4.111558	H	-3.665297	2.922468	-3.835003
H	0.279815	2.287434	-2.769823	H	1.1684084	3.3417707	-2.3728052
H	1.266495	0.239872	-4.219044	H	1.3098743	1.2474278	-4.0435371
H	3.924788	0.181849	-3.905615	H	3.7988934	0.2214977	-3.9018790
H	4.592166	2.140653	-2.193452	H	5.1638996	1.6399556	-2.0909425
H	2.346382	3.439473	-1.487898	H	3.5799198	3.6045350	-1.1771727
H	-5.041706	3.626281	3.395452	H	-6.669714	2.441100	3.323927
H	-4.968552	3.862797	1.647844	H	-6.767522	2.608197	1.568025
H	-6.180061	2.788532	2.337189	H	-7.240906	1.094495	2.334822
H	-8.841172	-0.306509	-0.098530	H	-8.535648	-1.939349	-1.002429
H	-7.879366	0.743267	0.946049	H	-8.203496	-0.718214	0.233632
H	-7.932991	1.040845	-0.788518	H	-7.891780	-0.362106	-1.462197
H	1.136682	2.371059	2.990478	H	2.3500433	3.0198102	2.3646987
H	1.404424	-1.621790	1.458471	H	0.6736519	-0.9235879	2.1879704
H	-1.831644	2.119736	-0.007148	H	-1.787696	2.294694	0.547253

H	2.098679	3.465903	1.023385	H	2.0910354	4.0948116	0.3256957
H	1.743386	1.546770	5.218681	H	3.1514593	2.5691837	4.6319233
H	1.995875	-2.447910	3.686426	H	1.4984209	-1.3789502	4.4403231
H	-2.659029	4.427472	0.112223	H	-2.817006	4.511963	0.676442
H	4.174481	2.550183	0.096156	H	4.8535872	2.2066687	0.2352840
H	4.688890	-0.785992	2.725281	H	4.7002484	-1.3546268	2.6027662
H	6.332957	-1.653372	1.004382	H	5.5590552	-2.7761251	0.4199119
H	2.327626	-2.737258	-0.051273	H	1.3798725	-1.8792760	0.5876134
H	6.561706	1.365699	-0.081637	H	6.8327891	0.0340525	-0.0047295
H	4.920985	-1.807544	-2.449940	H	4.0489542	-1.9321386	-2.6017112
H	2.162031	-0.862791	5.591849	H	2.7384460	0.3681284	5.6905894
H	4.695637	4.087581	1.940488	H	6.1397181	3.3342051	2.0037329
H	5.167620	0.744243	4.572651	H	5.9549632	-0.2203231	4.3821735
H	6.719878	-3.917424	1.860562	H	5.0916973	-5.0689570	1.1471069
H	2.714700	-5.005435	0.797135	H	0.9087123	-4.1588132	1.3371490
H	8.557278	1.398385	-1.511110	H	8.6660609	-0.5470280	-1.5239496
H	6.929079	-1.783717	-3.870413	H	5.8938103	-2.5477570	-4.1056944
H	5.189900	3.192407	4.199413	H	6.6870544	2.1256601	4.0965293
H	4.911924	-5.610547	1.772110	H	2.7622093	-5.7867152	1.5903905
H	8.754676	-0.169462	-3.416490	H	8.2115936	-1.8424621	-3.5889547
H	-5.092557	4.181606	-2.910144	H	-6.178467	3.429330	-2.998389
H	-6.234647	3.112877	-2.091045	H	-6.927502	1.931991	-2.437284
H	-5.093289	4.060377	-1.147250	H	-6.419996	3.147308	-1.269965
H	-1.114357	6.273091	0.706887	H	-1.397311	6.547501	0.639449
H	1.267296	5.776563	1.167140	H	1.0603173	6.3213488	0.4715923
Br	-2.253933	-2.990277	1.292624	Br	-1.754818	-2.448862	1.617741

Table S9. Cartesian coordinates of the geometry optimized structures of $\mathbf{3}\boldsymbol{u}^{+}$ in the gas phase; b3lyp (left) and PBE0 (right), def2-TZVP/ECP(W,Ru).

Ru	2.836546	0.979429	-1.080523	Ru	2.8267871	0.9767807	-1.0395414
C	3.881291	2.988430	-0.909630	C	3.8421512	2.9531357	-0.9505624
C	4.824021	1.970039	-0.617523	C	4.7450313	1.9646946	-0.4747391
C	3.451293	2.827957	-2.250842	C	3.5505229	2.6700835	-2.3058555
C	4.135965	1.699602	-2.799399	C	4.2711218	1.4970503	-2.6785733
C	4.982134	1.172994	-1.794933	C	5.0027556	1.0625763	-1.5493257
P	1.199893	1.504194	0.483233	P	1.1886151	1.5853063	0.4709298
P	3.265357	-1.124114	-0.106394	P	3.0781940	-1.1369929	-0.0271012
S	1.061851	0.470648	-2.439439	S	1.0813202	0.6014112	-2.4397100
W	-2.213445	0.240731	-0.733033	W	-2.212631	0.383197	-0.786983
C	-2.144449	-0.042161	-2.713079	C	-2.124280	0.258056	-2.766228
C	-0.331611	0.742862	-0.079529	C	-0.342472	0.849301	-0.104967
C	-0.292990	0.435321	-1.384091	C	-0.295695	0.579316	-1.420515
N	-2.120876	-1.930345	-0.377776	N	-2.044902	-1.788262	-0.598942
N	-2.867842	0.523203	1.411619	N	-2.822030	0.586759	1.362621
N	-4.377369	-0.406106	-0.921497	N	-4.323744	-0.376048	-0.890811
C	-2.715833	1.500344	2.321399	C	-2.687256	1.535850	2.296540
N	-3.488186	-0.519067	2.047056	N	-3.234680	-0.538655	1.994900
C	-3.718979	-0.209125	3.338946	C	-3.341111	-0.324365	3.317464
B	-4.044136	-1.686728	1.238347	B	-3.793021	-1.698942	1.182252
C	-5.357959	-0.190147	-1.820136	C	-5.329041	-0.246471	-1.767910
N	-4.921555	-1.143164	0.099117	N	-4.771766	-1.154932	0.129839
C	-6.225340	-1.373405	-0.144109	C	-6.049458	-1.507545	-0.088799
C	-1.466751	-2.936690	-0.997510	C	-1.434706	-2.726893	-1.346001
N	-2.906614	-2.501418	0.591226	N	-2.685890	-2.435650	0.409119
C	-2.748855	-3.838116	0.585564	C	-2.472859	-3.756983	0.321369
O	-2.059407	-0.168289	-3.849904	O	-2.033186	0.262604	-3.908365
C	1.474517	1.249255	2.249692	C	1.4586429	1.3835319	2.2396184
C	0.820561	3.291088	0.344380	C	0.8103478	3.3661584	0.3118265
C	2.314043	-1.743598	1.324775	C	2.1453731	-1.6912695	1.4413850
C	5.009243	-1.258376	0.425411	C	4.8118342	-1.4089491	0.4701243
C	3.078409	-2.489987	-1.308299	C	2.7856485	-2.5053213	-1.1962050
C	-2.097636	2.826830	2.067250	C	-2.233154	2.924295	2.053432
C	-3.229078	1.065741	3.548914	C	-2.999339	0.991914	3.545788
C	-4.396615	-1.126606	4.297211	C	-3.733743	-1.376243	4.288615
C	-5.193676	0.527694	-3.113505	C	-5.230377	0.486074	-3.053964
C	-6.534790	-0.777688	-1.354354	C	-6.437534	-0.939449	-1.286898
C	-7.110749	-2.138361	0.777407	C	-6.844706	-2.344269	0.844394
C	-0.528393	-2.779351	-2.140491	C	-0.696827	-2.492027	-2.608318
C	-1.821660	-4.143483	-0.397339	C	-1.657744	-3.974057	-0.772288
C	-3.481625	-4.757460	1.499224	C	-3.038507	-4.750275	1.263351
C	2.705185	1.619982	2.796377	C	2.6766710	1.7970890	2.7770390
C	0.479818	0.738482	3.077234	C	0.4617844	0.9120519	3.0813790
C	2.933022	1.490980	4.158157	C	2.8885392	1.7483535	4.1443626

C	1.933544	0.983521	4.982637	C	1.8849972	1.2826316	4.9840949
C	0.711787	0.607084	4.439631	C	0.6773154	0.8626783	4.4497602
C	0.281457	3.773127	-0.851018	C	0.3967907	3.8548043	-0.9270243
C	1.082535	4.184023	1.383946	C	0.9062687	4.2406966	1.3909586
C	0.007007	5.123995	-0.999980	C	0.0833077	5.1932731	-1.0806918
C	0.279747	6.012108	0.034480	C	0.1834245	6.0620836	-0.0027212
C	0.817717	5.538865	1.224455	C	0.5931002	5.5825009	1.2303115
C	5.471972	-0.470642	1.484051	C	5.3867867	-0.5841415	1.4382448
C	5.904429	-2.104762	-0.227565	C	5.5767695	-2.4208107	-0.1016699
C	7.237617	-2.156328	0.166680	C	6.8988680	-2.5988563	0.2832686
C	7.687789	-1.373636	1.220662	C	7.4629008	-1.7778997	1.2457860
C	6.798345	-0.532987	1.883063	C	6.7027020	-0.7699427	1.8264244
C	3.232352	-2.252024	-2.674022	C	3.0289482	-2.3232536	-2.5546842
C	2.833473	-3.793247	-0.868727	C	2.4564415	-3.7774403	-0.7287683
C	2.733286	-4.833033	-1.782856	C	2.3856537	-4.8473827	-1.6067669
C	2.879284	-4.585690	-3.143137	C	2.6452342	-4.6613844	-2.9560924
C	3.132858	-3.293397	-3.586710	C	2.9612952	-3.3961004	-3.4298178
C	2.891434	-2.074463	2.550458	C	2.7701185	-1.9970693	2.6487741
C	0.943671	-1.937750	1.149791	C	0.7702486	-1.8917643	1.3250133
C	2.106250	-2.579125	3.579786	C	2.0340623	-2.4765466	3.7212987
C	0.743414	-2.767585	3.394938	C	0.6674103	-2.6660045	3.5994323
C	0.162827	-2.445350	2.174959	C	0.0403911	-2.3776907	2.3971028
H	3.536658	3.750816	-0.231579	H	3.4375437	3.7768287	-0.3826461
H	5.363915	1.844849	0.303136	H	5.2037098	1.9373313	0.4989595
H	5.642592	0.328944	-1.897462	H	5.6538758	0.2034425	-1.5099458
H	4.033275	1.328616	-3.805805	H	4.2678639	1.0347356	-3.6538441
H	2.726019	3.441408	-2.757691	H	2.8796274	3.2327264	-2.9368164
H	-2.870397	3.589577	1.962612	H	-3.089790	3.593337	1.950058
H	-4.586085	1.422906	-3.009167	H	-4.621045	1.383669	-2.964220
H	-4.682739	-2.397089	1.942563	H	-4.340721	-2.467548	1.911789
H	-1.459898	3.104308	2.906839	H	-1.634716	3.266265	2.899395
H	-1.513129	2.838116	1.159134	H	-1.647902	3.009829	1.144080
H	-3.246100	1.622891	4.469791	H	-2.967615	1.502035	4.494921
H	-4.388139	-0.684563	5.291815	H	-3.494877	-1.041191	5.297925
H	-5.435130	-1.305800	4.015023	H	-4.803039	-1.598047	4.247176
H	-3.900273	-2.096571	4.347912	H	-3.201111	-2.309506	4.100200
H	-6.173740	0.815840	-3.490033	H	-6.225890	0.778805	-3.388351
H	-4.728052	-0.116482	-3.861131	H	-4.793525	-0.152632	-3.825928
H	-7.493460	-0.766611	-1.843128	H	-7.401985	-1.026294	-1.760187
H	-8.123421	-2.152397	0.379373	H	-7.705088	-2.761469	0.321811
H	-6.776206	-3.170095	0.894055	H	-6.257746	-3.163295	1.259133
H	-7.140204	-1.687109	1.769966	H	-7.216104	-1.748312	1.682541
H	-0.126778	-3.754085	-2.408512	H	-0.312203	-3.445034	-2.969205
H	-1.027949	-2.364537	-3.014202	H	-1.351561	-2.077008	-3.374527
H	0.312844	-2.132076	-1.909701	H	0.1490793	-1.8170409	-2.4903112
H	-1.451875	-5.120216	-0.656439	H	-1.283443	-4.919226	-1.130121
H	-3.087154	-5.766261	1.393581	H	-2.583537	-5.722491	1.077474
H	-3.380632	-4.454057	2.541534	H	-2.847402	-4.473656	2.302208

H	-4.547543	-4.783225	1.268212	H	-4.118945	-4.849902	1.134479
H	3.478279	2.020320	2.153431	H	3.4497459	2.1800302	2.1201654
H	-0.466455	0.436819	2.657282	H	-0.481919	0.584896	2.668661
H	3.885776	1.786588	4.577644	H	3.8309743	2.0854848	4.5593364
H	2.110551	0.879406	6.044928	H	2.0501882	1.2485097	6.0545297
H	-0.066513	0.204494	5.074094	H	-0.111826	0.499209	5.097331
H	0.051383	3.096275	-1.660064	H	0.2971572	3.1839986	-1.7715134
H	1.475691	3.828291	2.324550	H	1.2044891	3.8767622	2.3650819
H	-0.434978	5.478731	-1.921292	H	-0.259176	5.556422	-2.042092
H	0.064147	7.065685	-0.083368	H	-0.073787	7.107590	-0.123692
H	1.022773	6.221947	2.038298	H	0.6609392	6.2514111	2.0800858
H	4.788233	0.183837	2.005851	H	4.7888519	0.1888646	1.9069292
H	5.567499	-2.727242	-1.043616	H	5.1446358	-3.0771454	-0.8456052
H	7.921445	-2.815928	-0.350967	H	7.4849724	-3.3888990	-0.1704486
H	8.723974	-1.418611	1.528119	H	8.4908588	-1.9294622	1.5526991
H	7.139687	0.074509	2.711119	H	7.1359973	-0.1295571	2.5854803
H	3.418425	-1.248461	-3.025998	H	3.2791643	-1.3377722	-2.9271370
H	2.714373	-3.997055	0.185486	H	2.2670509	-3.9387854	0.3247892
H	2.539860	-5.837812	-1.430802	H	2.1369356	-5.8317898	-1.2280089
H	2.795430	-5.396640	-3.854511	H	2.6013185	-5.5017879	-3.6386194
H	3.247411	-3.093731	-4.643803	H	3.1631817	-3.2447280	-4.4834543
H	3.951483	-1.955555	2.709045	H	3.8393980	-1.8864610	2.7588748
H	0.481055	-1.692047	0.210432	H	0.2624183	-1.6852602	0.3928293
H	2.566861	-2.827234	4.526779	H	2.5385018	-2.7119490	4.6507561
H	0.135922	-3.159702	4.200069	H	0.0921093	-3.0436328	4.4368450
H	-0.897486	-2.573536	2.021624	H	-1.025452	-2.522122	2.288268
Br	-3.102616	2.611792	-1.143601	Br	-3.209738	2.692416	-1.127969

Table S10. Cartesian coordinates of the geometry optimized structures of virtual **3*u***$^+$-W(III) in the gas phase; b3lyp (left) and PBE0 (right), def2-TZVP/ECP(W,Ru).

Ru	2.871800	0.979705	-1.031420	Ru	2.8058438	0.8061727	-1.0710690
C	3.841851	3.005475	-0.966918	C	3.8079858	2.7688214	-1.0994032
C	4.825112	2.023298	-0.663994	C	4.7633584	1.7781393	-0.7475750
C	3.366501	2.783030	-2.282269	C	3.3225656	2.4951732	-2.4023654
C	4.074842	1.652007	-2.808485	C	3.9881618	1.3169939	-2.8659608
C	4.974871	1.194711	-1.821670	C	4.8759415	0.8831640	-1.8579143
P	1.214990	1.534922	0.449973	P	1.1740463	1.5080120	0.3582070
P	3.300620	-1.086999	-0.098851	P	3.2588908	-1.1814649	-0.0086659
S	1.073744	0.229995	-2.426726	S	1.0097309	-0.0036217	-2.3877942
W	-2.198519	0.181869	-0.729739	W	-2.263056	0.233400	-0.721618
C	-2.096900	-0.323449	-2.733775	C	-2.261070	-0.071976	-2.761457
C	-0.347459	0.761382	-0.127938	C	-0.399828	0.742465	-0.156211
C	-0.244112	0.349447	-1.405446	C	-0.313919	0.249551	-1.410134
N	-2.128092	-1.959574	-0.255955	N	-2.315501	-1.939357	-0.497350
N	-2.867119	0.627769	1.337039	N	-2.782905	0.549757	1.397581
N	-4.384125	-0.446936	-0.904470	N	-4.472610	-0.255770	-0.798178
C	-2.760222	1.694253	2.152761	C	-2.542013	1.507952	2.304499
N	-3.478843	-0.368432	2.055506	N	-3.267484	-0.528759	2.069219
C	-3.739505	0.059133	3.306511	C	-3.307497	-0.272357	3.386899
B	-4.068980	-1.576046	1.332384	B	-4.054905	-1.580903	1.292391
C	-5.355160	-0.304468	-1.829695	C	-5.509489	0.121097	-1.563500
N	-4.948199	-1.081112	0.172599	N	-4.986192	-0.858431	0.307154
C	-6.253138	-1.314688	-0.059130	C	-6.327475	-0.821379	0.272212
C	-1.486584	-3.022185	-0.800990	C	-1.781994	-2.976882	-1.179970
N	-2.961456	-2.452844	0.720726	N	-3.108503	-2.470159	0.475458
C	-2.836947	-3.789364	0.796609	C	-3.059080	-3.808099	0.431835
O	-1.977086	-0.572957	-3.833159	O	-2.203416	-0.191154	-3.884337
C	1.388431	1.327725	2.241407	C	1.3640567	1.4009856	2.1493560
C	0.788950	3.310620	0.274544	C	0.7604822	3.2613072	0.0258643
C	2.361394	-1.685825	1.361665	C	2.2292907	-1.8802057	1.3341282
C	5.045307	-1.225867	0.446719	C	4.9059406	-1.0558425	0.7638967
C	3.122847	-2.527068	-1.223878	C	3.4114475	-2.6595010	-1.0730965
C	-2.197382	3.011235	1.770332	C	-2.042022	2.867248	2.007986
C	-3.287786	1.362496	3.402134	C	-2.855041	1.017888	3.571687
C	-4.406424	-0.781724	4.338667	C	-3.744768	-1.272608	4.390297
C	-5.170302	0.264094	-3.193446	C	-5.398600	0.701992	-2.924123
C	-6.544315	-0.827504	-1.321789	C	-6.694038	-0.203434	-0.908754
C	-7.154292	-1.982895	0.920126	C	-7.190791	-1.335173	1.363042
C	-0.530197	-2.976606	-1.938841	C	-0.968278	-2.901770	-2.416403
C	-1.899721	-4.177672	-0.147180	C	-2.209545	-4.161681	-0.598887
C	-3.599261	-4.634607	1.755698	C	-3.800344	-4.696278	1.355298
C	2.604970	1.701628	2.817627	C	2.4566590	2.0466127	2.7320637
C	0.363833	0.855194	3.053790	C	0.5296280	0.6243760	2.9417891
C	2.784074	1.623348	4.190475	C	2.6993918	1.9262890	4.0890831

C	1.751943	1.157869	4.999212	C	1.8652089	1.1399442	4.8749826
C	0.547088	0.770358	4.427681	C	0.7833517	0.4933871	4.2983866
C	0.324789	3.755696	-0.966449	C	0.4327484	3.5927806	-1.2891961
C	0.959971	4.231179	1.307559	C	0.8042849	4.2687777	0.9845622
C	0.023567	5.094717	-1.163161	C	0.1671877	4.9040008	-1.6413885
C	0.201355	6.010124	-0.131098	C	0.2256041	5.9064767	-0.6832764
C	0.674121	5.575717	1.100715	C	0.5431270	5.5843851	0.6269357
C	5.511339	-0.384112	1.461587	C	5.0581000	-0.2032727	1.8568044
C	5.939687	-2.111157	-0.152779	C	6.0323632	-1.6618631	0.2183188
C	7.271261	-2.149824	0.249669	C	7.2929425	-1.3938717	0.7390946
C	7.721934	-1.315391	1.262632	C	7.4356243	-0.5353549	1.8174327
C	6.834487	-0.433844	1.872692	C	6.3101513	0.0477501	2.3881681
C	3.236140	-2.339975	-2.600706	C	3.4470036	-2.5334639	-2.4574658
C	2.923886	-3.819318	-0.732234	C	3.5151830	-3.9294440	-0.5053278
C	2.829478	-4.896844	-1.602713	C	3.6414572	-5.0486860	-1.3092276
C	2.933470	-4.698001	-2.975623	C	3.6584854	-4.9156531	-2.6921014
C	3.141226	-3.417342	-3.472709	C	3.5648552	-3.6573221	-3.2651278
C	2.928645	-1.941936	2.608487	C	2.7196477	-2.2241854	2.5904454
C	0.995281	-1.910153	1.190710	C	0.8929796	-2.1425982	1.0398199
C	2.142288	-2.401316	3.659289	C	1.8806724	-2.7814918	3.5445658
C	0.785065	-2.622758	3.475364	C	0.5490260	-3.0184079	3.2494042
C	0.212373	-2.376313	2.233613	C	0.0579936	-2.7055739	1.9897212
H	3.504266	3.784855	-0.304556	H	3.4974715	3.5976378	-0.4812218
H	5.410890	1.959973	0.234744	H	5.3396082	1.7353382	0.1613698
H	5.653920	0.364403	-1.916304	H	5.5291728	0.0266771	-1.9139256
H	3.949904	1.234251	-3.793979	H	3.8440590	0.8490177	-3.8282300
H	2.624097	3.366333	-2.798036	H	2.6061273	3.0823899	-2.9526371
H	-3.001194	3.697599	1.497172	H	-2.873928	3.576401	2.002080
H	-4.574871	1.172697	-3.187177	H	-4.568968	1.397949	-3.019700
H	-4.711322	-2.225348	2.088380	H	-4.688340	-2.258836	2.042384
H	-1.657942	3.441694	2.612559	H	-1.343448	3.180302	2.784177
H	-1.527680	2.944179	0.925850	H	-1.550635	2.930198	1.044157
H	-3.333155	2.001920	4.266519	H	-2.745627	1.544266	4.505680
H	-4.406604	-0.256172	5.291546	H	-3.540541	-0.893106	5.390872
H	-5.441428	-0.999036	4.070813	H	-4.811974	-1.492807	4.315889
H	-3.894728	-1.735481	4.471156	H	-3.206360	-2.213256	4.261747
H	-6.144531	0.494965	-3.620474	H	-6.315320	1.236978	-3.171708
H	-4.687649	-0.458335	-3.854384	H	-5.274911	-0.093988	-3.664083
H	-7.498420	-0.849670	-1.819032	H	-7.695029	-0.011022	-1.259284
H	-8.166867	-2.010153	0.522910	H	-8.230238	-1.099877	1.138014
H	-6.840206	-3.007791	1.122220	H	-7.101420	-2.416713	1.485886
H	-7.174935	-1.450200	1.871616	H	-6.934568	-0.868851	2.316662
H	0.120138	-3.847681	-1.898872	H	-0.246867	-3.719848	-2.428907
H	-1.065997	-3.007820	-2.889946	H	-1.621145	-3.025960	-3.285480
H	0.099022	-2.095124	-1.943616	H	-0.413096	-1.977293	-2.527727
H	-1.548861	-5.176957	-0.336704	H	-1.931414	-5.157091	-0.904785
H	-3.230257	-5.657204	1.711195	H	-3.457082	-5.720921	1.219450
H	-3.495820	-4.273257	2.778857	H	-3.646273	-4.413011	2.397789

H	-4.663699	-4.645836	1.517156	H	-4.873647	-4.665026	1.152667
H	3.406349	2.058400	2.183301	H	3.1265452	2.6361726	2.1147075
H	-0.567665	0.539136	2.613325	H	-0.306241	0.106154	2.493826
H	3.727064	1.921029	4.630060	H	3.5444580	2.4383971	4.5339552
H	1.891935	1.090383	6.069996	H	2.0694998	1.0255531	5.9330557
H	-0.254150	0.393170	5.049470	H	0.1323654	-0.1267639	4.9031043
H	0.191755	3.054320	-1.777381	H	0.3917804	2.8152666	-2.0424127
H	1.305254	3.903989	2.277328	H	1.0367208	4.0321380	2.0148569
H	-0.353105	5.423032	-2.122827	H	-0.093961	5.142299	-2.665682
H	-0.028302	7.055813	-0.287072	H	0.0192990	6.9338496	-0.9590248
H	0.816054	6.282518	1.907887	H	0.5870322	6.3619992	1.3803673
H	4.830400	0.308988	1.935362	H	4.1874336	0.2786208	2.2838816
H	5.603983	-2.773570	-0.937297	H	5.9316479	-2.3352953	-0.6248879
H	7.953782	-2.838948	-0.230208	H	8.1644970	-1.8601210	0.2946327
H	8.756585	-1.349569	1.576951	H	8.4188530	-0.3188808	2.2184431
H	7.176260	0.217467	2.666793	H	6.4107520	0.7098595	3.2403363
H	3.395436	-1.344397	-2.989187	H	3.3767127	-1.5471421	-2.8997059
H	2.841742	-3.985942	0.332494	H	3.4928580	-4.0441101	0.5720517
H	2.678318	-5.893481	-1.208393	H	3.7262932	-6.0288487	-0.8547610
H	2.856595	-5.538032	-3.653492	H	3.7504923	-5.7940564	-3.3199894
H	3.226674	-3.255143	-4.539134	H	3.5853771	-3.5484023	-4.3431494
H	3.985568	-1.797275	2.768684	H	3.7612209	-2.0656560	2.8352670
H	0.544656	-1.720753	0.231818	H	0.5079774	-1.9135587	0.0537015
H	2.598375	-2.588182	4.622566	H	2.2782596	-3.0379666	4.5195470
H	0.176489	-2.980163	4.295816	H	-0.102604	-3.455967	3.996849
H	-0.844419	-2.534000	2.081372	H	-0.977471	-2.901670	1.746014
Br	-2.982691	2.450197	-1.496593	Br	-2.946022	2.559365	-1.302321
